# Automated acquisition of explainable knowledge from unannotated histopathology images

**DOI:** 10.1038/s41467-019-13647-8

**Published:** 2019-12-18

**Authors:** Yoichiro Yamamoto, Toyonori Tsuzuki, Jun Akatsuka, Masao Ueki, Hiromu Morikawa, Yasushi Numata, Taishi Takahara, Takuji Tsuyuki, Kotaro Tsutsumi, Ryuto Nakazawa, Akira Shimizu, Ichiro Maeda, Shinichi Tsuchiya, Hiroyuki Kanno, Yukihiro Kondo, Manabu Fukumoto, Gen Tamiya, Naonori Ueda, Go Kimura

**Affiliations:** 1 Pathology Informatics Team, RIKEN Center for Advanced Intelligence Project, Nihonbashi 1-chome Mitsui Bldg. 15F, 1-4-1 Nihonbashi, Chuo-ku, Tokyo, 103-0027 Japan; 2 Department of Pathology, Shinshu University School of Medicine, 3-1-1 Asahi, Matsumoto, Nagano, 390-8621 Japan; 3 Department of Surgical Pathology, Aichi Medical University Hospital, 1-1 Yazakokarimata, Nagakute, Aichi, 480-1195 Japan; 4 Department of Urology, Nippon Medical School Hospital, 1-1-5 Sendagi, Bunkyo-ku, Tokyo, 113-8603 Japan; 5 Statistical Genetics Team, RIKEN Center for Advanced Intelligence Project, Nihonbashi 1-chome Mitsui Bldg. 15F, 1-4-1 Nihonbashi, Chuo-ku, Tokyo, 103-0027 Japan; 6 Department of Urology, St. Marianna University School of Medicine, 2-16-1 Sugao, Miyamae-ku, Kawasaki, Kanagawa, 216-8511 Japan; 7 Department of Analytic Human Pathology, Nippon Medical School, 1-1-5 Sendagi, Bunkyo-ku, Tokyo, 113-8603 Japan; 8 Department of Pathology, St. Marianna University School of Medicine, 2-16-1 Sugao, Miyamae-ku, Kawasaki, Kanagawa, 216-8511 Japan; 9 Diagnostic Pathology, Ritsuzankai Iida Hospital, 1-15 Odori, Iida, Nagano, 395-8505 Japan; 10 Institute of Development, Aging and Cancer, Tohoku University, 4-1 Seiryo-machi, Aoba-ku, Sendai, Miyagi, 980-8575 Japan; 11 Tohoku Medical Megabank Organization, Tohoku University, 2-1 Seiryo-machi, Aoba-ku, Sendai, Miyagi, 980-8573 Japan; 12 Goal-Oriented Technology Research Group, RIKEN Center for Advanced Intelligence Project, Nihonbashi 1-chome Mitsui Bldg. 15F, 1-4-1 Nihonbashi, Chuo-ku, Tokyo, 103-0027 Japan

**Keywords:** Cancer imaging, Translational research, Computer science

## Abstract

Deep learning algorithms have been successfully used in medical image classification. In the next stage, the technology of acquiring explainable knowledge from medical images is highly desired. Here we show that deep learning algorithm enables automated acquisition of explainable features from diagnostic annotation-free histopathology images. We compare the prediction accuracy of prostate cancer recurrence using our algorithm-generated features with that of diagnosis by expert pathologists using established criteria on 13,188 whole-mount pathology images consisting of over 86 billion image patches. Our method not only reveals findings established by humans but also features that have not been recognized, showing higher accuracy than human in prognostic prediction. Combining both our algorithm-generated features and human-established criteria predicts the recurrence more accurately than using either method alone. We confirm robustness of our method using external validation datasets including 2276 pathology images. This study opens up fields of machine learning analysis for discovering uncharted knowledge.

## Abstract (web summary)

Technologies for acquiring explainable features from medical images need further development. Here, the authors report a deep learning based automated acquisition of explainable features from pathology images, and show a higher accuracy of their method as compared to pathologist based diagnosis of prostate cancer recurrence.

## Introduction

Trained on massive amounts of annotated data, deep learning algorithms have been successfully used in medical image classification and cancer detection. Esteva et al. successfully used a deep neural network to categorize fine-grained images of skin tumors, including malignant melanomas, at a dermatologist level^1^. Fauw et al. detected a range of sight-threatening retinal diseases as efficiently as an expert ophthalmologist, even on a clinically heterogeneous set of three-dimensional optical coherence tomographs (OCTs)^2^. Chilamkurthy et al. retrospectively collected a large annotated dataset of head computed tomography (CT) and evaluated the potential of deep learning algorithm to identify critical findings on CT images^3^. Bejnordi et al. evaluated the performance of deep learning algorithms submitted as part of a challenge competition and found that the performance of the high-ranking algorithm was comparable to that of pathologists in the detection of breast cancer metastases in histopathological tissue sections of lymph nodes^4^. Currently, machine learning-enhanced hardware is also being developed. Google has announced the development of an augmented reality microscope based on deep learning algorithms to assist pathologists^5^.

Pathological examinations are used to provide definitive diagnoses and are one of the most reliable examinations in current cancer medicine^6^, but diagnostic pathology knowledge and skills can only be acquired by completing a long fellowship program^7^. Although machine learning-driven histopathological image analysis^4,8,9^ is an attractive tool for assisting doctors, it faces two significant hurdles: the need for explainable analyses to gain clinical approval and the tremendous amount of information in histopathological images^8,10^. Acquiring explainable knowledge from medical images is imperative for medicine. Furthermore, there are substantial size differences between histopathological images and other medical images^1–3,11,12^. A pathology slide contains large number of cells and the image consists of 10-billion-scale pixels^8^.

We develop a method to acquire explainable features from diagnostic annotation-free histopathological images and assess the prediction accuracy of prostate cancer recurrence using our algorithm-generated features by comparison with that of human-established cancer criteria, the Gleason score, provided by expert pathologists in the diagnosis of prostate cancer.

## Results

### Key feature generation

**Fig. 1:**
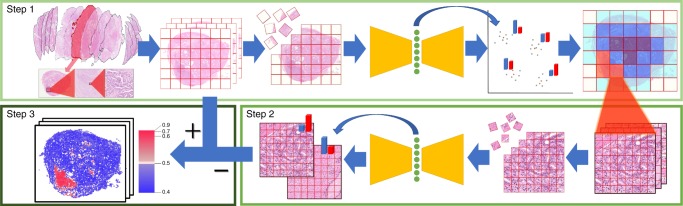
Key feature generation method. This method is a type of dimensionality reduction that emphasizes not only the nucleus structure examined at high magnification but also the structural pattern examined at low magnification. Step 1: First, we divide low-magnification pathology images into smaller images, then perform dimensionality reduction using a deep autoencoder followed by weighted non-hierarchical clustering. This process reduces an image with 10-billion-scale pixel data to only 100 feature data with scores. Step 2: Next, we analyze high-magnification images in order to reduce the number of misclassified low-magnification images. Again, we divide these into smaller images, before applying a second deep autoencoder and calculating average scores for the images. Step 3: Results of Step 2 complementarily correct those of Step 1. We remove images in which the results of Steps 1 and 2 do not match. Finally, we use the total numbers of each type of feature to make predictions, for example, to make cancer recurrence predictions, create human-understandable features or automatically annotate images. The color of each region indicates positive (red) and negative (blue) for characteristics detected.

**Fig. 2:**
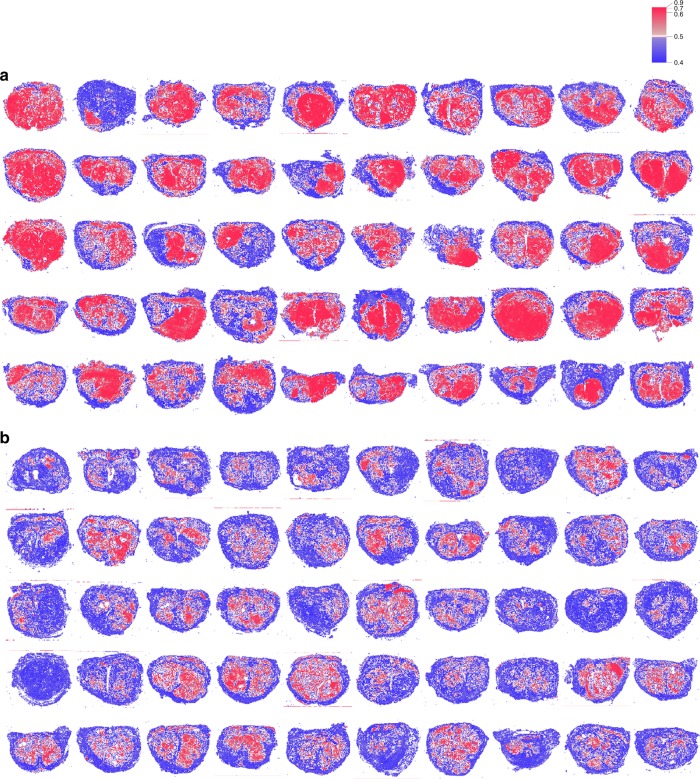
Examples of compressed images. Whole-mount pathology images with 10-billion-scale pixel data were reduced to only 100 feature data, while retaining core image information. **a** Images of biochemical recurrence (BCR) cases, **b** images of no BCR cases. The color of each region indicates positive (red) and negative (blue) for characteristics detected.

First, we have developed a method of generating key features that employs two different unsupervised deep neural networks (deep autoencoders^13,14^) at different magnifications and weighted non-hierarchical clustering^15^ (Fig. 1 and Supplementary Figs. 1 and 2). This takes histopathological images with 10-billion-scale pixel data and reduces them to only 100 feature data with scores while retaining the images’ core information (see Key feature generation method in the Methods section and Fig. 2). This method is a type of dimensionality reduction and was inspired by the complementary diagnostic process of pathologists that emphasizes not only the nucleus structure examined at high magnification but also the structural pattern examined at low magnification. These features can be effective for tasks such as predicting cancer recurrence, understanding the contributions of particular features and automatically annotating images. In the key feature generation dataset, short-term biochemical recurrence (BCR) cases from Nippon Medical School Hospital (NMSH) were considered positive purely based on the recurrence time for patients (the recurrence period range: 1.7–14.4 months). No direct information regarding cancer images was provided to deep neural networks.

### BCR predictions for evaluation of generated features

Next, we evaluated the accuracy of cancer recurrence prediction using deep learning-generated features by comparing the predicted results with that using the Gleason score, one of the most crucial clinicopathological factors in the current prostate cancer practice^16^. The main purpose of the Gleason score is to predict prognosis. The Gleason grading system defines five architectural growth patterns, which provides information on prostate cancer aggressiveness and facilitates patients’ appropriate care. As prostate cancer usually harbors two or more Gleason patterns, the sum of primary and secondary patterns yields the Gleason score. In this paper, all images’ Gleason scores were evaluated by expert pathologists.

We conducted two evaluations: BCR predictions based on the cross-validation method using dataset from NMSH excluding 100 cases that were used for key feature generation, and external validation of BCR predictions based on datasets from St. Marianna University Hospital (SMH) and Aichi Medical University Hospital (AMH).

**Fig. 3:**
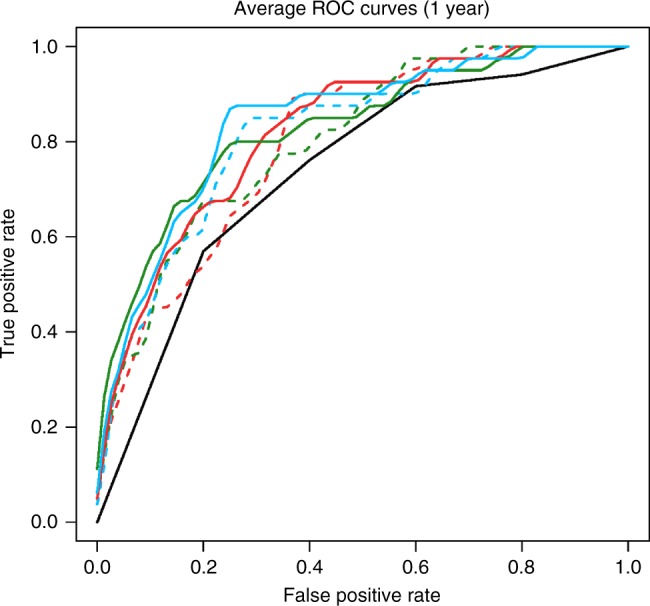
Biochemical recurrence (BCR) prediction. Average receiver operating characteristic (ROC) curves for the BCR prediction within one year. Gleason score (black solid line), Ridge (red dot line), Lasso (green dot line), support vector machine (SVM; blue dot line), Ridge + Gleason score (red solid line), Lasso + Gleason score (green solid line), SVM + Gleason score (blue solid line).

NMSH dataset included a total of 13,188 whole-mount pathology images from 1007 patients who received radical prostatectomy (Supplementary Fig. 3). We excluded 115 cases involving neoadjuvant therapy and 7 cases involving adjuvant therapy, as well as 43 cases who could not be followed up within 1 year because of hospital transfer or death due to other causes, thus leaving 842 cases for analysis (Supplementary Fig. 4a). Table 1 and Supplementary Table 1 summarize the clinical characteristics of NMSH dataset. Cancer was more likely to recur in patients with higher prostate-specific antigen (PSA) levels (*P*-value < 0.001, Wilcoxon rank sum test). It was more likely to recur in patients with higher Gleason scores (≥8) than in patients with lower Gleason scores (<8). Similar patterns were observed for 1-year and 5-year recurrence rates. No significant differences existed for the average age, height, weight, or prostate weight between patients in whom cancer recurred and those in whom it did not. We categorized the data for 842 patients into the following two sets: 100 patients (100 whole-mount pathology images, the largest among available images per each patient) were used to generate key features using the deep neural networks, and 742 (9816 whole-mount pathology images) were used to perform the BCR predictions using these features (Supplementary Fig. 4a). We applied lasso^17^ and ridge^18^ regression analyses and a support vector machine (SVM)^19^ to the features to predict the BCR of prostate cancer. We evaluated the areas under the receiver operating characteristic curves (AUCs) with a 95% confidence interval (CI), receiver operating characteristic (ROC) curves^20,21^ and pseudo *R*-squared values^22^. Table 2 and Fig. 3 present the AUCs, pseudo *R*-squared values and ROC curves of BCR prediction using the deep learning-generated features and we compared these values to those of the Gleason score. The AUC for BCR prediction based on the deep neural networks within 1 year was 0.820 (95% CI: 0.766–0.873), while that on the Gleason score was 0.744 (95% CI: 0.672–0.816). Interestingly, combining both methods produced a more accurate BCR prediction [AUC, 0.842 (95% CI: 0.788–0.896)] than either method alone. Likewise, the 5-year prediction accuracies were 0.721 (95% CI: 0.672–0.769; deep neural networks), 0.695 (95% CI: 0.639–0.750; Gleason score), and 0.758 (95% CI: 0.710–0.806; both criteria combined) (Supplementary Table 2 and Supplementary Fig. 5). We also obtained the highest pseudo *R*-squared values by combining Gleason score and criteria generated by deep neural networks, followed by that based on deep neural network predictions alone then that based on Gleason score alone.

**Table 1:** The clinical characteristics of Nippon Medical School Hospital (NMSH) dataset.

	BCR^a^ (within 1 year), *n* = 79	No BCR^a^ (within 1 year), *n* = 763	*P*-value
Mean age, years (S.D.^b^, range)	66.8 (5.7, 53–76)	66.7 (6.0, 41–81)	0.94
Mean height, cm (S.D.^b^, range)	165 (7.0, 147–185)	166 (5.7, 150–194)	0.17
Mean weight, kg (S.D.^b^, range)	65.4 (10, 42–96)	65.0 (11, 40–193)	0.99
Gleason score (≥8)/*n* (%)	57/79 (72%)	231/763 (30%)	5.2 × 10^–13^
Mean PSA^c^, ng/mL (S.D.^b^, range)	24.5 (27, 4.3–165)	11.7 (15, 0.6–218.9)	1.1 × 10^–13^
Mean prostate weight, g (S.D.^b^, range)	49.2 (21, 11–142)	45.9 (17, 10–138)	0.060
Clinical recurrence/*n* (%)	14/79 (18%)	9/763 (1%)	5.5 × 10^–10^

We compared the characteristics of patients whose cancer did or did not recur using the Fisher’s exact test for categorical data and the Wilcoxon rank-sum test for continuous data

^a^Biochemical recurrence (BCR)

^b^Standard deviation (S.D.)

^c^Prostate-specific antigen (PSA)

**Table 2:** Comparison of biochemical recurrence (BCR) within 1 year.

	AUC^a^	Pseudo *R*-squared
Gleason score (pathologist)	0.744 [95% CI 0.672–0.816]	0.182 [*p*-value 0.11]
Ridge (automated)	0.801 [95% CI 0.748–0.854]	0.222 [*p*-value 0.018]
Lasso (automated)	0.804 [95% CI 0.749–0.860]	**0.227 [*p*-value 0.018]**
SVM (automated)	**0**.**820 [95% CI 0**.**766–0**.**873]**	0.194 [*p*-value 0.090]
Ridge + Gleason score	0.824 [95% CI 0.770–0.878]	0.267 [*p*-value 0.0098]
Lasso + Gleason score	0.830 [95% CI 0.772–0.888]	**0.334 [*p*-value 0.0060]**
SVM + Gleason score	**0**.**842 [95% CI 0**.**788–0**.**896]**	0.247 [*p*-value 0.037]

BCR predictions at Nippon Medical School Hospital (NMSH) (cross validation)

AUC^a^: the reported values are averages with 95% confidence interval. The bold values indicate the highest accuracies for lasso, ridge and support vector machine (SVM)

^a^Area under the curve (AUC)

As external validation of BCR predictions, we applied our method and the 100 features derived from training data from NMSH to the datasets from two other hospitals: SMH and AMH, in total 102 patients who received radical prostatectomy. These cases corresponded to a total of 2276 pathology images. We excluded 1 case involving neoadjuvant therapy and 1 case because of missing slides, as well as 5 cases who could not be followed up within 1 year because of hospital transfer, thus leaving 95 cases for analysis (Supplementary Fig. 4b). Supplementary Table 3 summarizes the clinical characteristics of the validation datasets. Only PSA level of patients with recurrent cancer at SMH was significantly higher than that of non-recurrent patients (*P*-value = 0.0043, Wilcoxon rank sum test). In external validation, the 1-year prediction accuracies were 0.845 (95% CI: 0.761–0.928; deep neural networks), 0.721 (95% CI: 0.552–0.889; Gleason score), and 0.884 (95% CI: 0.782–0.985; both criteria combined) (Table 3 and Supplementary Fig. 6). Similar tendency was observed in AUCs and pseudo *R*-squared values for prediction of cancer recurrence. The results of external validation indicate robust applicability of our method and rule out overfitting that is often encountered in deep neural networks studies. Supplementary Tables 4 and 5 show comparison of BCR for each hospital (SMH and AMH) independently.

**Table 3:** Comparison of biochemical recurrence (BCR) within 1 year.

	AUC^a^	Pseudo *R*-squared
Gleason score (pathologist)	0.721 [95% CI 0.552–0.889]	0.110 [*p*-value 0.030]
Ridge (automated)	0.829 [95% CI 0.743–0.915]	0.244 [*p*-value 0.0010]
Lasso (automated)	0.810 [95% CI 0.700–0.921]	0.207 [*p*-value 0.0026]
SVM (automated)	**0.845 [95% CI 0.761–0.928]**	**0.260 [*p*-value 0.00069]**
Ridge + Gleason score	0.871 [95% CI 0.782–0.960]	0.310 [*p*-value 0.00019]
Lasso + Gleason score	**0.884 [95% CI 0.782–0.985]**	0.294 [*p*-value 0.00029]
SVM + Gleason score	0.882 [95% CI 0.803–0.962]	**0.319 [*p*-value 0.00015]**

BCR predictions at St. Marianna University Hospital (SMH) and Aichi Medical University Hospital (AMH) (external validation)

The reported values are averages with 95% confidence interval. The bold values indicate the highest accuracies for lasso, ridge and support vector machine (SVM)

^a^Area under the curve (AUC)

### Explainable features from histopathology images

**Fig. 4:**
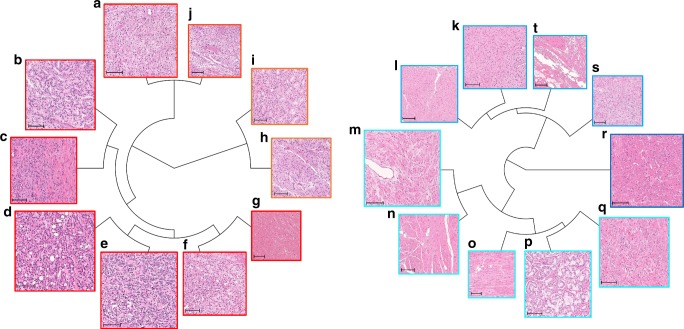
Representative images of key features. The top 10 images are closest to the centroids of the 100 features, with higher-ranking images being larger, in the biochemical recurrence (BCR) group (**a**–**j**) and no BCR group (**k**–**t**). **a**–**j** Cancers equivalent to Gleason patterns 4 or 5, which usually indicate aggressive clinical behavior. **c** Dense stromal components without cancer cells. **g** Hemorrhage. **p** Cancers equivalent to Gleason pattern 3, which usually indicates benign clinical behavior. **k**–**o**, **q**–**s** Loose stromal components without cancer cells. **t** Surgical margin without cancer cells. The scale bar included in each image represents a length of 100 μm. Expert genitourinary pathologist’s comments on BCR images (**a**–**j**): Cancers show Gleason patterns 4 or 5 indicating aggressive clinical behavior. Stromal component without cancer cells tends to show dense cellularity compared to those of normal structure. The pathologist’s comments on no BCR images (**k**–**t**): Cancers show Gleason pattern 3 indicating indolent clinical behavior. Stromal component without cancer cells tends to show relatively loose cellularity suggesting normal peripheral zone structure. Cauterized extraprostatic connective tissue without cancer cells, which indicate that the surgical margin is free from cancer.

**Fig. 5:**
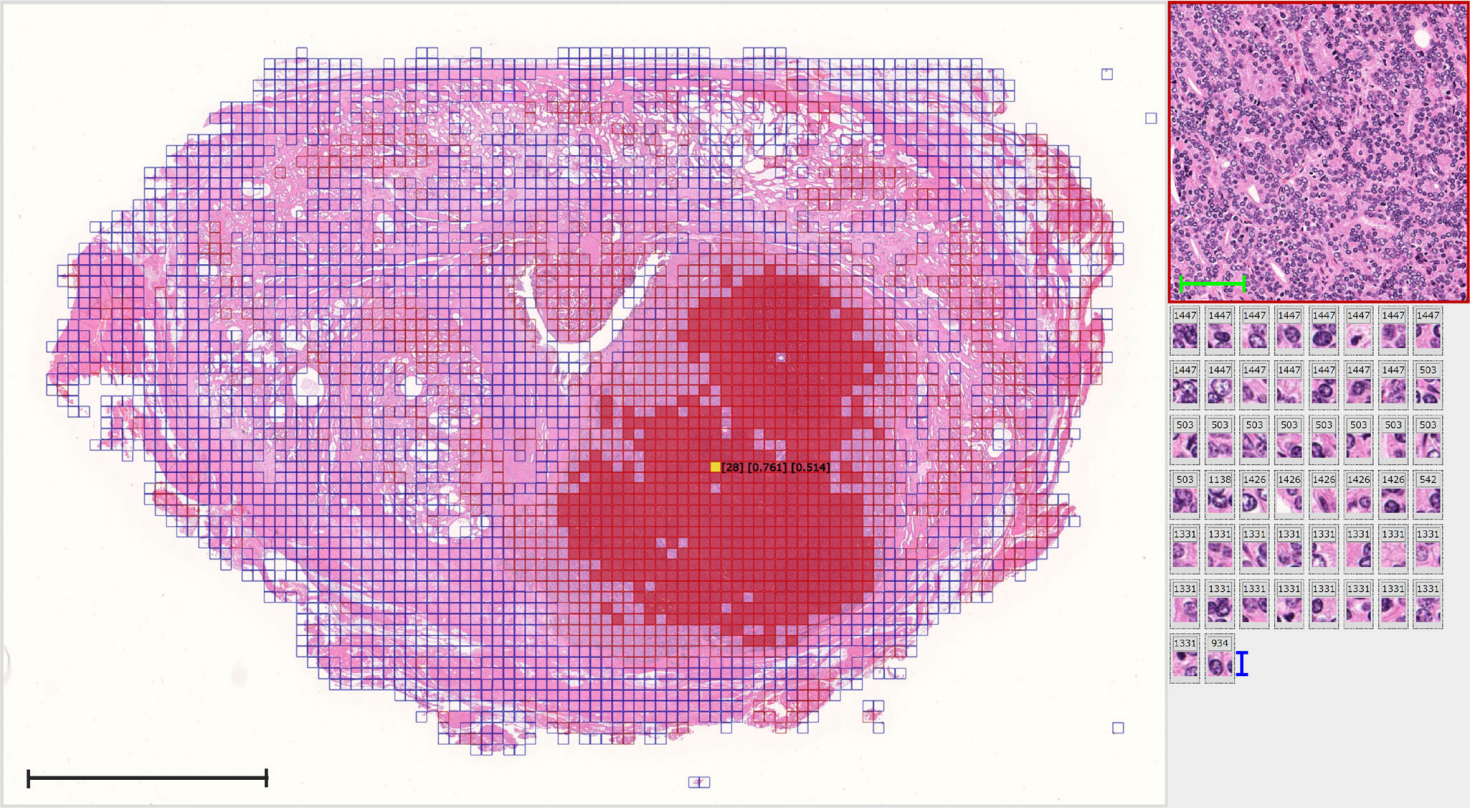
Automatically annotated whole-mount pathology image. Our method directly generates key features based on the whole image without requiring a region selection step. Using the key features and cell-level information found by the deep neural networks, we automatically annotated whole-mount pathology images. Here we show an automatically annotated whole-mount pathology image (left), as well as a low-magnification image of the yellow region (upper right) and the associated high-magnification images with number of Step 2 feature (lower right). The regions with impact scores above and below 0.5 in Step 1 are shaded in red and blue, respectively. The indicated yellow cell shows [number of Step 1 feature (100 total features)] [impact score, Step 1] [impact score, Step 2] (see Key feature generation method in the Methods section). The black scale bar included in the image represents a length of 1 cm. The green scale bar represents a length of 100 μm. The blue scale bar represents a length of 12.3 μm.

We then selected the images that were closest to each centroid as being representative of the features (see Key feature generation method in the Methods section and Fig. 4). The expert GU pathologist (T. Tsuzuki, the second author) analyzed these images to search for pathological meanings (see figure legend in Fig. 4 and Supplementary Figs. 7–36). In summary, the pathologist found that the deep neural networks appeared to have mastered the basic concept of the Gleason score fully automatically, generating explainable key features that could be understood by pathologists. Furthermore, the deep neural networks identified the features of stroma in the noncancerous area as prognostic factors, which typically have not been evaluated in prostate histopathological images. Figure 5 and Supplementary Movies 1 and 2 show feature maps for a whole-mount pathology image, as well as cell-level information of images; the predicted high-grade cancer regions are shaded in red, whereas normal ducts/low-grade cancer regions are shaded in blue.

## Discussion

We achieved fully automated acquisition of explainable features from histopathological images in the raw. Pathologists can understand these features in terms of histopathology because the images described in this study were large enough for pathologists to understand and included not only the nucleus structure examined at high magnification but also the structural pattern examined at low magnification. Our method found not only human-established findings but also previously-unrecognized pathological features, resulting in higher prediction accuracy of cancer recurrence than that of diagnosis performed by expert pathologists using human-established cancer criteria, the Gleason score.

The Gleason score^23^ is a unique pathological grading system, purely based on architectural disorders, without considering cytological atypia. In this study, none of the cancer cells in the images identified by the deep neural networks as representative of high-grade cancer showed severe nuclear atypia or prominent nucleoli. Our results indicate that the central ideas behind Gleason’s grading system are sound.

The most accurate BCR predictions were produced by combining the deep learning-generated features and Gleason score, possibly because the automatically derived features included factors different from those used for the Gleason score, such as the surgical margin status. Various and complex factors are believed to be associated with BCR^24,25^. Interestingly, representative images of the features nominated by the deep neural networks comprised of not only human-established findings but also previously unspotlighted or neglected features of stroma at the noncancerous area. These findings indicate that the deep neural networks could explore unique features that could be underestimated or overlooked by a human.

In this study, the deep neural networks identified comprehensible key features from scratch. Silver et al. reported that the AlphaGo Zero^26^ program, which is solely based on reinforcement learning without any human knowledge inputs, could defeat their previous AlphaGo^27^ program, which conducted supervised learning using human expert moves. In this study, we demonstrated another algorithm that performs well, is based on deep autoencoders^13,14^, and does not need human knowledge. Hopefully, this study will provide a tool for discovering uncharted findings. In addition, our method can be applied to non-verbal information, such as that derived from the subjective experience of experts, as long as it is used to classify images. For example, data from patients with similar symptoms but unknown causes could be used to discover the key underlying factors, resulting in more effective treatments and the development of medicines. We anticipate that our method will lead to innovative therapeutic strategies and will help reduce the workloads of busy physicians^28^. In the next step, we are planning to conduct clinical trials in order to confirm whether our method is universally effective for improving the prediction accuracy.

Human and computer analyses have different strengths. Our deep learning approach analyzes huge medical images broadly and without oversights or bias; human pathologists analyze the disease more accurately and with a greater focus on medical importance. Each approach can, therefore, complement the other. Medicine aims to save patients, and both medical doctors and artificial intelligence (AI) systems can contribute to this goal. The more effectively and deeply medical experts can utilize AI systems, the more patients will benefit. Increasing collaboration between medical experts and informaticians will surely improve outcomes for patients.

## Methods

### Data for key feature generation and BCR predictions (NMSH)

This hospital-based cohort comprised of all patients with prostate cancers who received radical prostatectomy from April 2000 to December 2016 at the NMSH (*N* = 1007). We collected whole-mount pathology slides and clinical data for all patients. Of note, no patients were enrolled on clinical trials of radical prostatectomy. We excluded 115 cases involving neoadjuvant therapy and 7 cases involving adjuvant therapy, as well as 43 cases who could not be followed up within 1 year because of hospital transfer or death due to other causes, thus leaving 842 cases for analysis (Supplementary Fig. 4a).

We categorized the data for 842 patients into the following two sets: 100 patients (100 whole-mount pathology images, the largest available image per each patient) were used to generate key features using the deep neural networks, and 742 (9816 whole-mount pathology images) were used to perform BCR predictions using these features (Supplementary Fig. 4a). We carefully ensured that no direct information regarding cancer concepts was provided to deep neural networks. In addition, histopathological images were not checked or annotated by pathologists before key feature generation was performed by the deep neural networks. In the key feature generation dataset, short-term BCR cases were considered positive purely based on the recurrence time for patients (the recurrence period range: 1.7–14.4 months). To avoid bias, we also used the same surgery year distribution to select negative cases. Of note, images that extended beyond the edge of the cover glass were not used for key feature generation. During the key feature generation process, we simply selected the largest available image per each patient without checking whether any cancer was included.

### Data for external validation of BCR predictions

This validation set comprised of all patients with prostate cancers who received radical prostatectomy from August 2013 to August 2017 at the SMH (*N* = 55) and from January 2016 to June 2016 at the AMH (*N* = 47). We collected whole-mount pathology slides and clinical data for all patients. No patients were enrolled on clinical trials of radical prostatectomy nor were part of the NMSH cohort. We combined both datasets of SMH and AMH (*N* = 102). We excluded 1 case involving neoadjuvant therapy and 1 case because of missing slides, as well as 5 cases who could not be followed up within 1 year because of hospital transfer, thus leaving 95 cases for analysis (Supplementary Fig. 4b).

### Ethics statements

This research has been approved by each Institutional Review Board (IRB): NMSH (reference 28-11-663), SMH (reference 3887), AMH (reference 2019-H045) and RIKEN (reference Wako3 29-14). It complies with all relevant ethical regulations. The informed consent was obtained in the form approved by each hospital IRB and the opportunity for refusal to participate in research has been guaranteed by an opt-out manner.

### Definition of BCR

We defined the BCR following radical prostatectomy based on the European Association of Urology guidelines of increasing PSA levels >0.2 ng/mL^29^. All patients were followed and checked for the BCR at the longest interval of every 3 months postoperatively; the follow-up duration was 72.8 ± 49.8 (mean ± standard deviation (S.D.)) months in the dataset from NMSH, 31.7 ± 17.8 (mean ± S.D.) months from SMH and 35.7 ± 9.51 (mean ± S.D.) months from AMH.

### Statistical analysis

We compared the characteristics of patients whose cancer did or did not recur using the Fisher’s exact test for categorical data and the Wilcoxon rank-sum test for continuous data (Table 1, Supplementary Tables 1 and 3). All tests were two-tailed and were considered statistically significant if *P*-value < 0.05. All statistical analyses were performed using R, version 3.4.4.

### Preparation of whole-mount pathology images

Whole prostates were fixed in 10% formalin and embedded in paraffin. All samples were sectioned at a thickness of 3 μm and stained with hematoxylin and eosin (H&E). All H&E-stained slides were scanned by a whole-slide imaging scanner (Hamamatsu NanoZoomer S60 Slide Scanner) with a ×20 objective lens and were stored on a secure computer.

### Histological grading

We classified prostate cancer histologically based on the International Society of Urological Pathologists (ISUP) classification criteria^16^. For NMSH cases, all slides were initially reviewed independently by two board-certified pathologists and our conclusions were confirmed by an expert pathologist (T.Tsuzuki) without using clinical data nor BCR data. For SMH and AMH cases, the Gleason score was provided independently by expert pathologists at each hospital without using clinical data nor BCR data.

### Key feature generation method

The proposed method does not require human annotation for image classification and reveals statistical distortions in image datasets by employing multiple deep autoencoders^13,14^ at different magnifications and weighted non-hierarchical clustering^15^. This takes histopathological images with 10-billion-scale pixel data and reduces them to only 100 feature data with scores while retaining the images’ core information. Supplementary Figs. 1 and 2 provide detailed algorithm flowcharts and descriptions of the autoencoder networks. We also evaluated 10, 50, 100 and 200 features (Supplementary Table 6). It was revealed that all these features showed almost same accuracies of BCR, but that 100 features set was the best. Previous methods include a region selection step, for example to extract or annotate the region of interest^30^. In contrast, our method derives the key features directly from the whole image, without requiring such a step. Our method is a type of dimensionality reduction and was inspired by the complementary diagnostic process of pathologists that emphasizes not only the nucleus structure examined at high magnification but also the structural pattern examined at low magnification.

In Step 1, we generated the key features from 100 whole-mount pathology images (100 cases), taken at low magnification (25×). We divided each pathology image (**S**_*i*_), into a set of 128 × 128-pixel image patches **S**_*i,j*_ using NDP.convert software (Hamamatsu Photonics K.K., version 2.0.7.0). Mean pixels of **S**_*i*_ was 145,025,449 ± 39,899,884 (mean ± S.D.). The number of images **S**_*i,j*_ per **S**_*i*_ was 8770 ± 2430 (mean ± S.D.). All these image patches completely cover the pathology images and were not allowed to overlap. We then applied a deep autoencoder we had developed for pathology images (Supplementary Fig. 2) to 128 × 128-pixel image patches, clustering the 2048 intermediate-layers to form 100 features (clusters) by k-means clustering. Features that included white background areas without tissue were automatically removed. Next, we found the centroid of each cluster generated by k-means, and calculated a score *u*_*i,j,k*_ (a score of *k*th feature in an image **S**_*i,j*_) based on the distance from each centroid *d*_*i,j.k*_. Here, we applied the simplest possible scoring method as follows:

1 $$\begin{array}{l}u_{i,j,k} = 1\ {\mathrm{if}}\ k = {\mathrm{argmin}}_k\,d_{i,j.k}\ {\mathrm{and}}\\ 0\ {\mathrm{otherwise}}\ \left( {k = 1,2, \ldots ,100} \right).\end{array}$$

Defining the total number of small images belonging to the positive and negative groups and *n*_positive_ and *n*_negative_, respectively, we defined the positive and negative degrees *r*_positive,*k*_ and *r*_negative,*k*_ for the *k*th feature as

2 $$r_{{\mathrm{positive}},k} = \varSigma _ + u_{i,j,k}/n_{{\mathrm{positive}}}\left( {k = 1,2, \ldots ,100} \right),$$

3 $$r_{{\mathrm{negative}},k} = \varSigma _ - u_{i,j,k}/n_{{\mathrm{negative}}}\left( {k = 1,2, \ldots ,100} \right),$$

where the sums *Σ*_*+*_ and *Σ*_−_ are over all *i,j* pairs such that image **S**_*i,j*_ belonged to the positive and negative groups, respectively. Finally, we defined the impact score *I*_*k*_ for the *k*th feature and the impact score *I*_*i,j*_ of image **S**_*i,j*_ for this step as

4 $$I_k = r_{{\mathrm{positive}},k}/\left( {r_{{\mathrm{positive}},k} + r_{{\mathrm{negative}},k}} \right)$$

5 $$I_{i,j} = \varSigma _kI_k \times u_{i,j,k}.$$

In Step 2, high-magnification (200×) images (**S***’*_*i*_) were analyzed to reduce the number of misclassified low-magnification images. Mean pixels of **S***’*_*i*_ was 9,281,628,733 ± 2,553,592,545 (mean ± S.D.). Here, 1024 × 1024-pixel image patches for each of 128 × 128-pixel image patches in Step 1 (**S***’*_*i,j*_) were divided into small 28 × 28-pixel image patches **S***’*_*i,j,j’*_ (the number of images **S***’*_*i,j,j’*_ per **S***’*_*i,j*_ was 1296). A second deep autoencoder (Supplementary Fig. 2) was then applied to each of 28 × 28-pixel image patches. The 1568 intermediate-layer features were given scores *u’*_*i,j,j’,k’*_ based on the intensity values ***v****’*_*i,j,j’*_ of each node. Again, we used the following simple scoring method:

6 $$\begin{array}{l}{u^\prime} _{i,j,j\prime ,k^\prime } = 1\ {\mathrm{if}}\,k^\prime = {\mathrm{argmax}}_{k^\prime }v^\prime _{i,j,j^\prime ,k^\prime }\ {\mathrm{and}}\\ 0\ {\mathrm{otherwise}}\ \left( {k^\prime = 1,2, \ldots ,1568} \right).\end{array}$$

Defining the total number of small images belonging to the positive and negative groups as *n’*_positive_ and *n’*_negative_, we defined the positive and negative degrees *r’*_positive,*k’*_ and *r’*_negative,*k’*_ for the *k’*th feature as

7 $$r^\prime _{{\mathrm{positive}},k\prime } = \varSigma _ + u^\prime _{i,j,j^\prime ,k^\prime }/n^\prime _{{\mathrm{positive}}}\left( {k^\prime = 1,2, \ldots ,1568} \right),$$

8 $${r^\prime} _{{\mathrm{negative}},k^\prime } = \varSigma _ - u^\prime _{i,j,j^\prime ,k^\prime }/n^\prime _{{\mathrm{negative}}}\left( {k^\prime = 1,2, \ldots ,1568} \right),$$

where the sums *Σ*_*+*_ and *Σ*_−_, analogously to those in Step 1, are over all *i,j,j’* such that the image **S***’*_*i,j,j’*_ belonged to the positive and negative groups, respectively. For this step, we defined the impact score *I’*_*i,j*_ as

9 $$\begin{array}{l}I^\prime _{i,j} =\ 	 \varSigma _{j^\prime }\varSigma _{k^\prime }\left( {r^\prime _{{\mathrm{positive}},k^\prime }/\left( {r^\prime _{{\mathrm{positive,}}k^\prime } + r^\prime _{{\mathrm{negative}},k^\prime }} \right)} \right)\\ 	\times u^\prime _{i,j,j^\prime ,k^\prime }/m,\end{array}$$

where *m* denotes the total number of small images **S***’*_*i,j,j’*_ used for **S**_*i,j*_.

In Step 3, results of Step 2 complementarily corrected those of Step 1. Images that were frequently in the positive and negative groups had impact scores above and below 0.5, respectively, so we defined images with impact scores above and below 0.5 as having positive and negative characteristics, respectively. We then removed images whose characters, based on the impact scores in Steps 1 and 2, did not match. Finally, we used the total numbers of each feature type for the subsequent predictions.

### Comparison of BCR predictions

To evaluate our approach, we predicted cancer recurrence using deep learning-generated 100 features. We used AUC and pseudo *R*-squared for comparison of deep learning-generated features and Gleason score^31^. AUC is the most frequently used metric to compare classifier performance, taking values ranging from 0 to 1. Higher the AUC, better the model is at classification. The pseudo *R*-squared value^22^ is a goodness-of-fit metric for regression models with a categorical response variable. It is an analogue to the *R*-squared for the ordinary least-squares regression and takes a value between 0 and 1. Higher the pseudo *R*-squared value, the better the model is at classification. We conducted two evaluations: BCR predictions based on cross validation using data at NMSH excluding 100 cases that were used for key feature generation, and external validation of BCR predictions based on data at SMH and AMH using the prediction model only trained by data at NMSH.

First, we predicted cancer recurrence using 9816 whole-mount pathology images (742 cases) at NMSH, excluding 100 cases that were used for key feature generation. In particular, we assessed the potential of the 100 features to predict the recurrence of cancer within 1 or 5 years postoperatively using Lasso^17^ and Ridge^18^ regression and a support vector machine (SVM)^19^, all popular methods for building prediction models. In addition, we created prediction models based on the application of logistic regression to an ISUP grade group assessed on the basis of the Gleason score and similarly created models combining the 100 features with the grade. If multiple images were available for a given patient, we averaged each feature over all the images. To address the fact that the feature values were not evenly distributed amongst patients where cancer did and did not recur, we multiplied each feature value by 1 + | *I*_*k*_–0.5| (see Key feature generation method in the Methods section), which augmented the predictive power of the models. We used 10-fold cross-validation^32,33^ to test the prediction models, randomly dividing the whole sample set in a 1: 9 ratio, using one part for testing and the other nine parts for training. In the cross-validation, the mean ratio of BCR cases to non-BCR cases within 1-year period was 4.00 ± 0.00 (mean ± S.D.): 70.2 ± 0.632 (mean ± S.D.). The ratio within 5-year period was 13.4 ± 1.26 (mean ± S.D.): 60.8 ± 0.632 (mean ± S.D.). For each testing/training split, we used the AUC and the pseudo *R*-squared metrics to assess the performance of trained prediction models on the test data^20,21^.

Next, using combined data of SMH and AMH, we predicted cancer recurrence using 2276 pathology images (95 cases) as external validation. We also assessed the potential of the same 100 features to predict the recurrence of cancer within 1 year postoperatively using the prediction models only trained by NMSH data. We also used the AUC and the *R*-squared metrics to assess the performance of trained prediction models on the test data^20,21^.

We determined hyperparameters for ridge (*λ*), lasso (*λ*) and SVM (*C* and *γ*) only within NMSH dataset (training data) by cross-validation. Those calculations were performed automatically using ready-made software packages. We used R for the analysis, using the glmnet package (version 2.0.16) for ridge and lasso regression, the e1071 package (version 1.7.0) for the SVM, and the cvAUC package (version 1.1.0) and pROC package (version 1.13.0) to evaluate the AUC with a CI. The pseudo *R*-squared were computed using rcompanion package (version 2.2.1).

### Reporting summary

Further information on research design is available in the Nature Research Reporting Summary linked to this Article.

## Supplementary Information

Supplementary Information

Description of Additional Supplementary Files

Supplementary Movie 1

Supplementary Movie 2

Reporting Summary

## Data Availability

The clinical datasets used were collected at the NMSH, SMH and AMH. This work and the collection of data were approved by the IRB of each hospital. They are not publicly available, and restrictions apply to their use. All the other data supporting the findings of this study are available within the Article and its Supplementary Information files. A Reporting Summary is available as a Supplementary Information file.

## Code availability

In the Key feature generation section, we presented our detailed algorithm flowchart, networks of deep autoencoders and the methods in this paper instead of full source code. All processes are described in enough detail to enable independent replication and the full source code were submitted for review process. All software (packages for R) in BCR predictions for evaluation of generated features are publicly available: the glmnet package (version 2.0.16), the e1071 package (version 1.7.0), cvAUC package (version 1.1.0), pROC package (version 1.13.0) and rcompanion package (version 2.2.1).

## References

[1] Esteva A (2017). Dermatologist-level classification of skin cancer with deep neural networks. Nature.

[2] De Fauw J (2018). Clinically applicable deep learning for diagnosis and referral in retinal disease. Nat. Med..

[3] Chilamkurthy S (2018). Deep learning algorithms for detection of critical findings in head CT scans: a retrospective study. Lancet.

[4] Ehteshami Bejnordi B (2017). Diagnostic assessment of deep learning algorithms for detection of lymph node metastases in women with breast cancer. JAMA.

[5] Chen PHC (2019). An augmented reality microscope with real-time artificial intelligence integration for cancer diagnosis. Nat. Med..

[6] Connolly, J. L. et al. in *Holland-Frei Cancer Medicine* 8th edn (ed. Hong, W. K.) 473–488 (PMPH-USA, Philadelphia, 2009).

[7] Barger LK (2005). Extended work shifts and the risk of motor vehicle crashes among interns. N. Engl. J. Med..

[8] Komura D, Ishikawa S (2018). Machine learning methods for histopathological image analysis. Comput. Struct. Biotechnol. J..

[9] Yamamoto Y (2017). Quantitative diagnosis of breast tumors by morphometric classification of microenvironmental myoepithelial cells using a machine learning approach. Sci. Rep..

[10] Gurcan MN (2009). Histopathological image analysis: a review. IEEE Rev. Biomed. Eng..

[11] Lakhani P, Sundaram B (2017). Deep learning at chest radiography: automated classification of pulmonary tuberculosis by using convolutional neural networks. Radiology.

[12] Kim K (2018). Performance of the deep convolutional neural network based magnetic resonance image scoring algorithm for differentiating between tuberculous and pyogenic spondylitis. Sci. Rep..

[13] Rumelhart, D. E., Hinton, G. E. & Williams, R. J. (eds) in *Parallel Distributed Processing* 318–362 (MIT Press, Cambridge, 1986).

[14] Hinton GE, Salakhutdinov RR (2006). Reducing the dimensionality of data with neural networks. Science.

[15] Arthur, D. & Vassilvitskii, S. k-means++: The advantages of careful seeding. In *Proc. of the Eighteenth Annual ACM-SIAM Symposium on Discrete Algorithms* (ed. Gabow, H.) 1027–1035 (Society for Industrial and Applied Mathematics, 2007).

[16] Epstein JI (2016). The 2014 international society of urological pathology (ISUP) consensus conference on Gleason grading of prostatic carcinoma: definition of grading patterns and proposal for a new grading system. Am. J. Surg. Pathol..

[17] Tibshirani R (1996). Regression shrinkage and selection via the Lasso. J. R. Stat. Soc. B.

[18] Hoerl AE, Kennard RW (1970). Ridge regression: biased estimation for nonorthogonal problems. Technometrics.

[19] Vapnik V (1998). Statistical Learning Theory. Ch.12.

[20] Pirracchio R (2015). Mortality prediction in intensive care units with the Super ICU Learner Algorithm (SICULA): a population-based study. Lancet Respir. Med..

[21] LeDell E, Petersen M, van der Laan M (2015). Computationally efficient confidence intervals for cross-validated area under the ROC curve estimates. Electron. J. Stat..

[22] Nagelkerke NJD (1991). A note on a general definition of the coefficient of determination. Biometrika.

[23] Phillips JL, Sinha AA (2009). Patterns, art, and context: Donald Floyd Gleason and the development of the Gleason grading system. Urology.

[24] Tsuzuki T (2015). Intraductal carcinoma of the prostate: a comprehensive and updated review. Int. J. Urol..

[25] Kato M (2019). Integrating tertiary Gleason pattern 5 into the ISUP grading system improves prediction of biochemical recurrence in radical prostatectomy patients. Mod. Pathol..

[26] Silver D (2017). Mastering the game of Go without human knowledge. Nature.

[27] Silver D (2016). Mastering the game of Go with deep neural networks and tree search. Nature.

[28] Robboy SJ (2013). Pathologist workforce in the United States: I. Development of a predictive model to examine factors influencing supply. Arch. Pathol. Lab. Med..

[29] Cornford P (2017). EAU-ESTRO-SIOG guidelines on prostate cancer. part II: treatment of relapsing, metastatic, and castration-resistant prostate cancer. Eur. Urol..

[30] Doyle S, Feldman M, Tomaszewski J, Madabhushi A (2012). A boosted Bayesian multiresolution classifier for prostate cancer detection from digitized needle biopsies. IEEE Trans. Biomed. Eng..

[31] Purcell SM (2009). Common polygenic variation contributes to risk of schizophrenia and bipolar disorder. Nature.

[32] Stone M (1974). Cross-validatory choice and assessment of statistical predictions. J. R. Stat. Soc. B..

[33] Hastie T, Tibshirani R, Friedman JH (2009). The Elements of Statistical Learning.

