# Supplementary material for: Automated acquisition of explainable knowledge from unannotated histopathology images
Source: Nat Commun. 2019 Dec 18;10:5642. doi: 10.1038/s41467-019-13647-8 (PMC6920352; doi:10.1038/s41467-019-13647-8)
Supplement: Supplementary file 1 — Supplementary Information [file 41467_2019_13647_MOESM1_ESM.pdf]

Automated acquisition of explainable knowledge from unannotated histopathology images

Yamamoto *et al.*

Supplementary Information

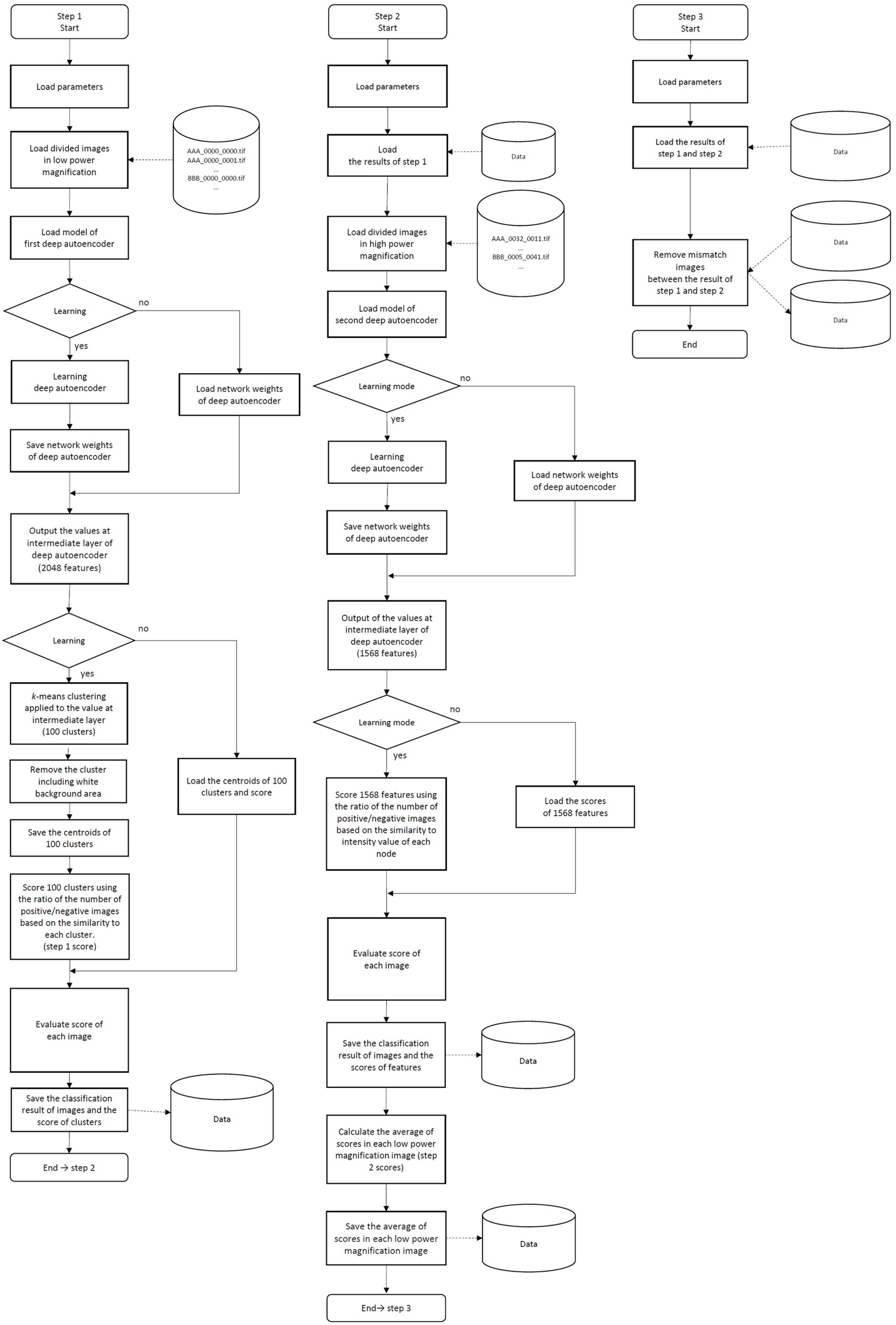

Supplementary Figure 1 | Algorithm flowcharts of key feature generation method.

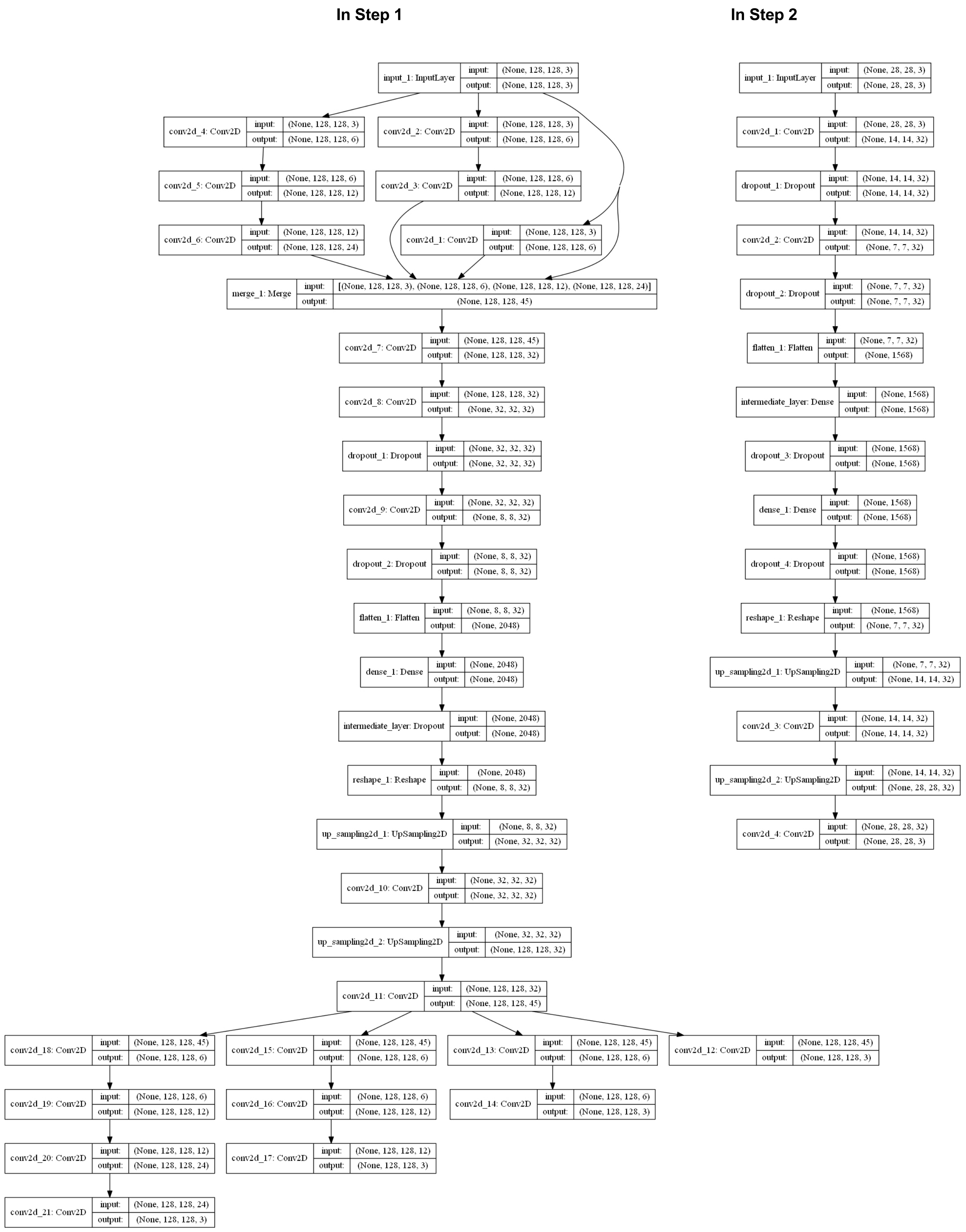

Supplementary Figure 2 | Networks of deep autoencoders.

Our method takes histopathological images with 10-billion-scale pixel feature data and reduces them to only 100 feature data with scores while retaining the images' core information. We applied two autoencoders at different magnifications.

In Step 1, 'intermediate-layer' included 2048 features. We applied k-means clustering to the 2048 features. As a result, we obtained 100 features.

The 100 features corresponded to 128×128-pixel patches in Step 1 and 1024×1024-pixel patches in Step 2.

All these patches completely covered the pathology images and were not allowed to overlap.

In Step 2, we obtained 'intermediate-layer' through dimensionality reduction of patches at high magnification.

The result of Step 1 was refined through the result of Step 2.

Each layer consists of layer name, tensor of shape (batch*, kernel height, kernel width, channels).

*Batch is shown as None in the figures because these figures only represent frameworks of the models.

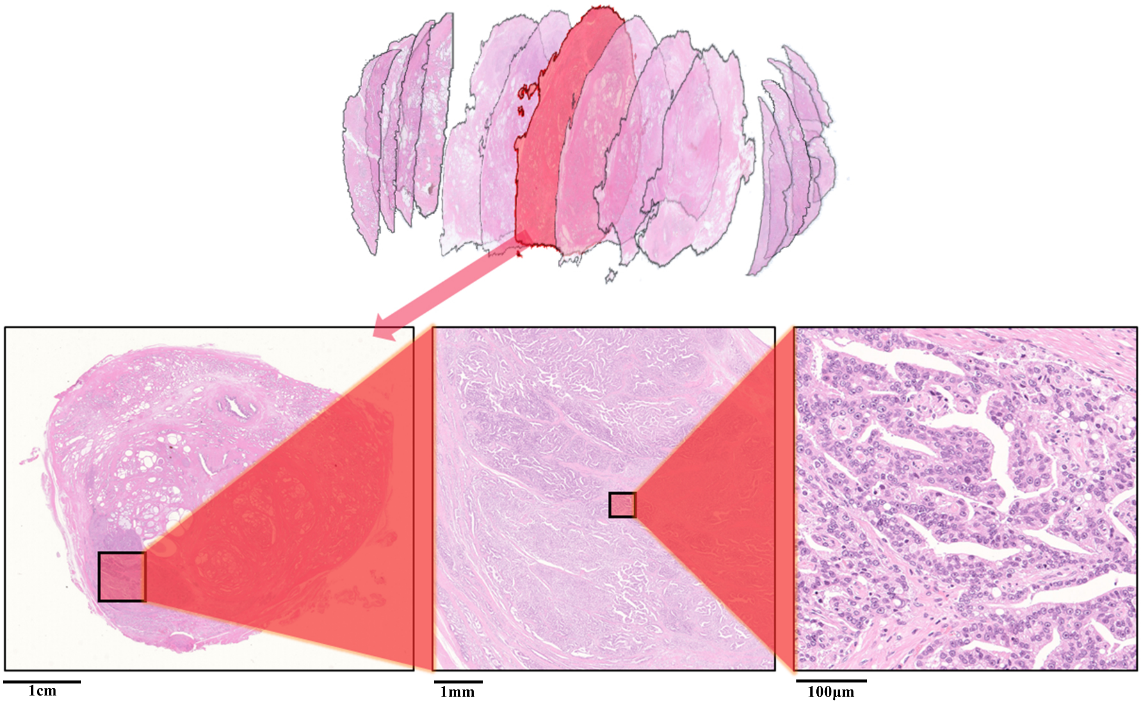

Supplementary Figure 3 | An example of a whole-mount pathology slide (118,272×99,456 pixels).

A set of whole-mount pathology images is highly informative medical data that contains pathological information of a whole organ. Formalin-fixed whole prostate was dissected into several approximately 3-5 mm slices and embedded in paraffin. All slices were further sectioned at a thickness of 3 µm and stained with H&E. All slides were digitalized. Each digital pathology image consisted of over several gigabytes.

Several whole-mount pathology images were obtained per each patient. We used the largest among available images per each patient for key feature generation. On the other hand, we used all whole-mount pathology images for cancer recurrence predictions.

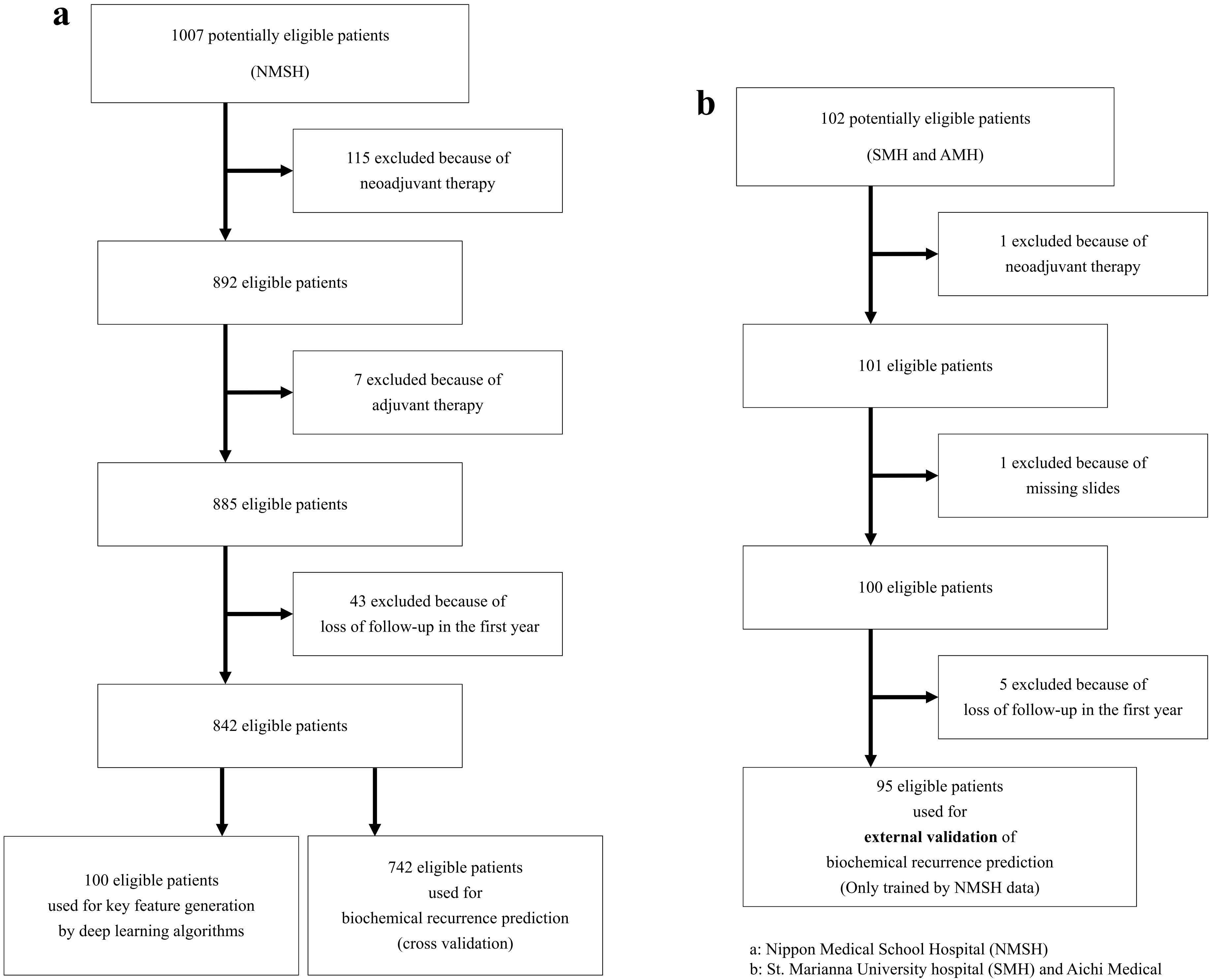

a: Nippon Medical School Hospital (NMSH)
b: St. Marianna University hospital (SMH) and Aichi Medical University Hospital (AMH) (external validation)

Supplementary Figure 4 | Dataset selection and validation process

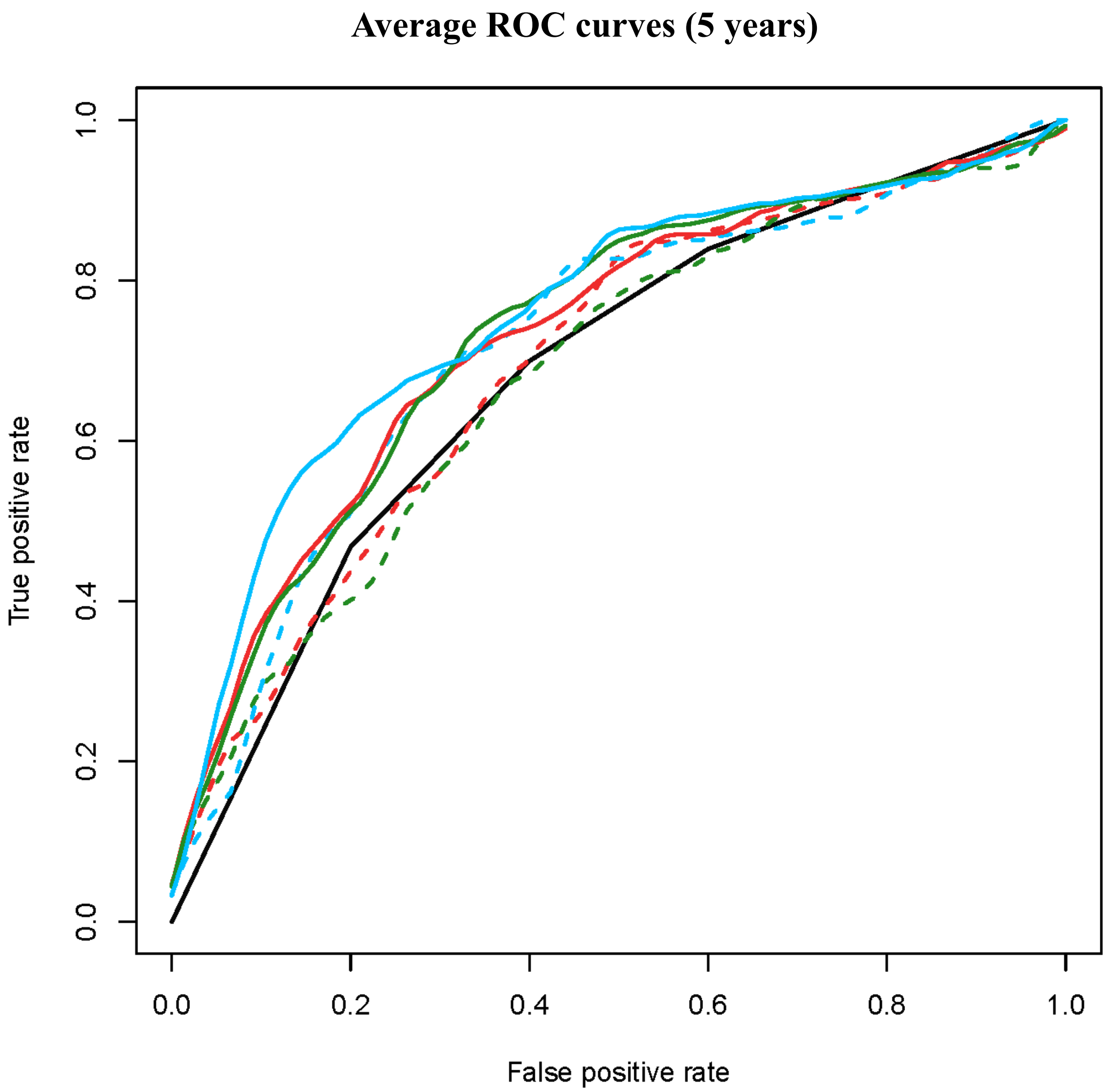

Supplementary Figure 5 | Biochemical recurrence (BCR) prediction within 5 years using data at Nippon Medical School Hospital (NMSH).

Average receiver operating characteristic (ROC) curves for the BCR prediction within five years. Gleason score (black solid line), Ridge (red dot line), Lasso (green dot line), support vector machine (SVM; blue dot line), Ridge + Gleason score (red solid line), Lasso + Gleason score (green solid line), SVM + Gleason score (blue solid line).

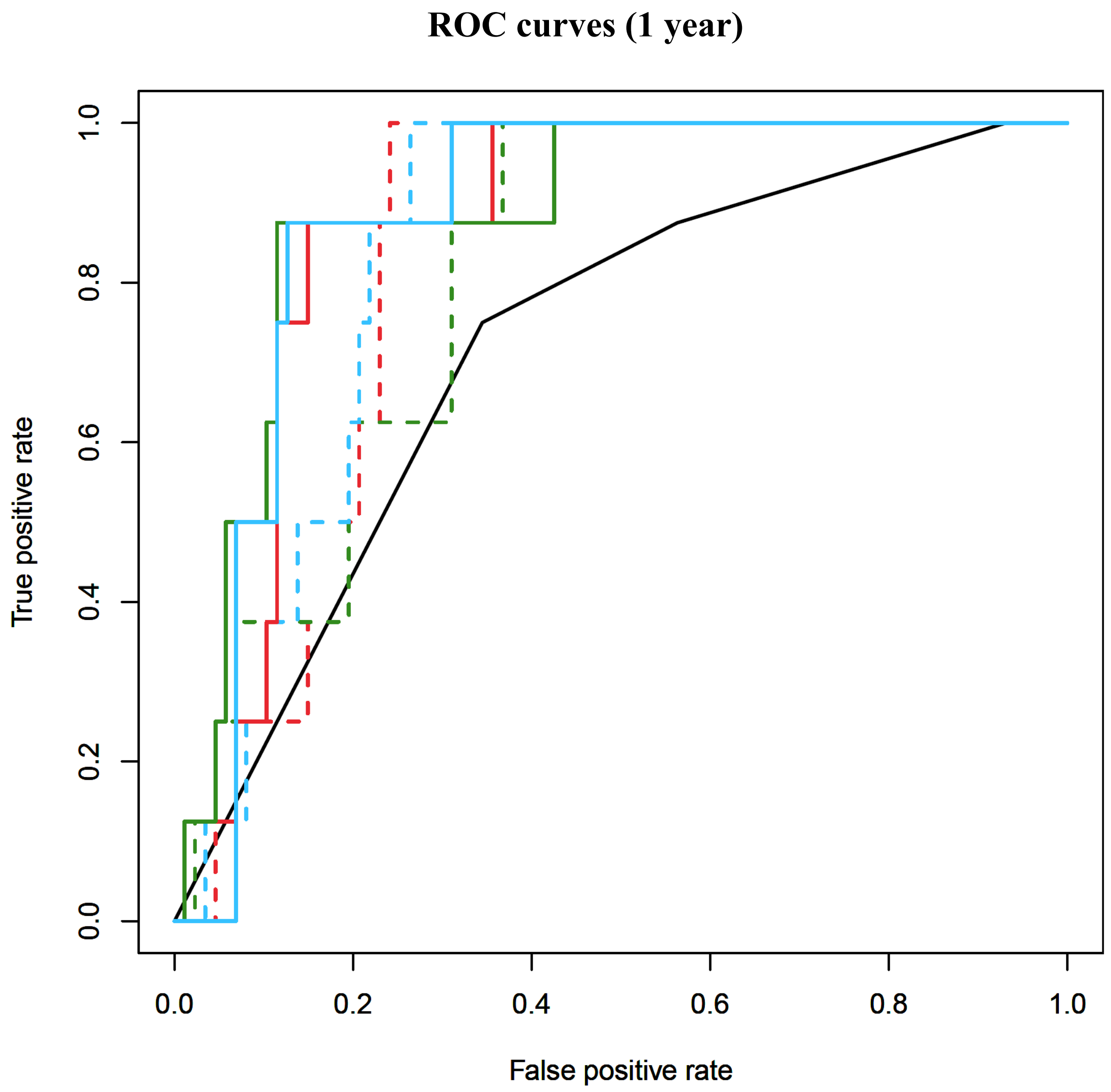

Supplementary Figure 6 | Biochemical recurrence (BCR) prediction within 1 year using data at St. Marianna University Hospital (SMH) and Aichi Medical University Hospital (AMH) (external validation).

Receiver operating characteristic (ROC) curves for the BCR prediction within one year. Gleason score (black solid line), Ridge (red dot line), Lasso (green dot line), support vector machine (SVM; blue dot line), Ridge + Gleason score (red solid line), Lasso + Gleason score (green solid line), SVM + Gleason score (blue solid line).

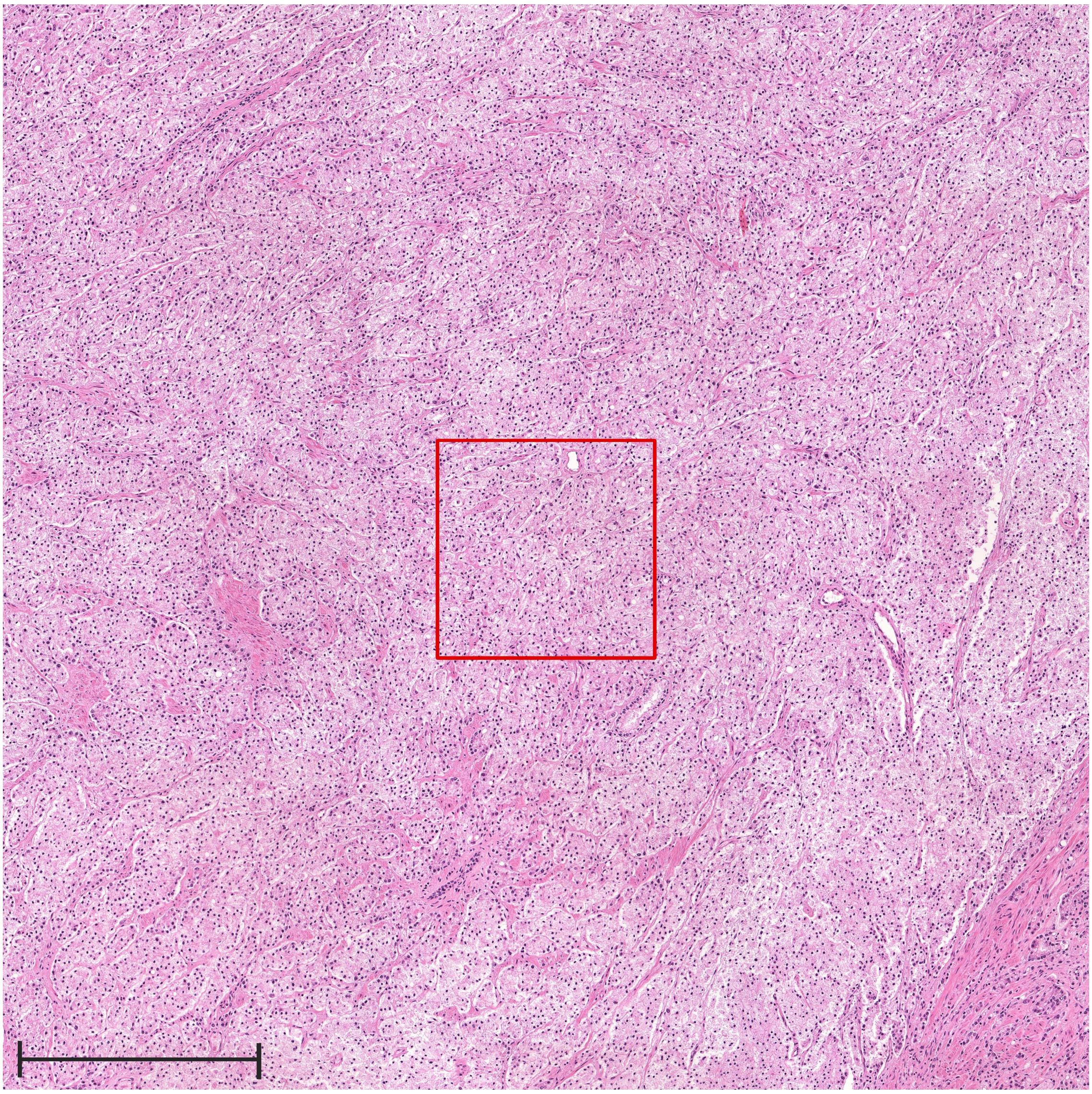

Supplementary Figure 7 | Representative image of a key feature (wide-range image).

Wide-range image contains a representative image from Figure 4a. The representative image was detected by our algorithm and is indicated with a red square. The scale bar included in the image represents a length of 500 μm. Expert genitourinary pathologist's comments: Adenocarcinoma. The area is entirely composed of adenocarcinoma cells without gland formation, which is diagnosed as Gleason pattern 5.

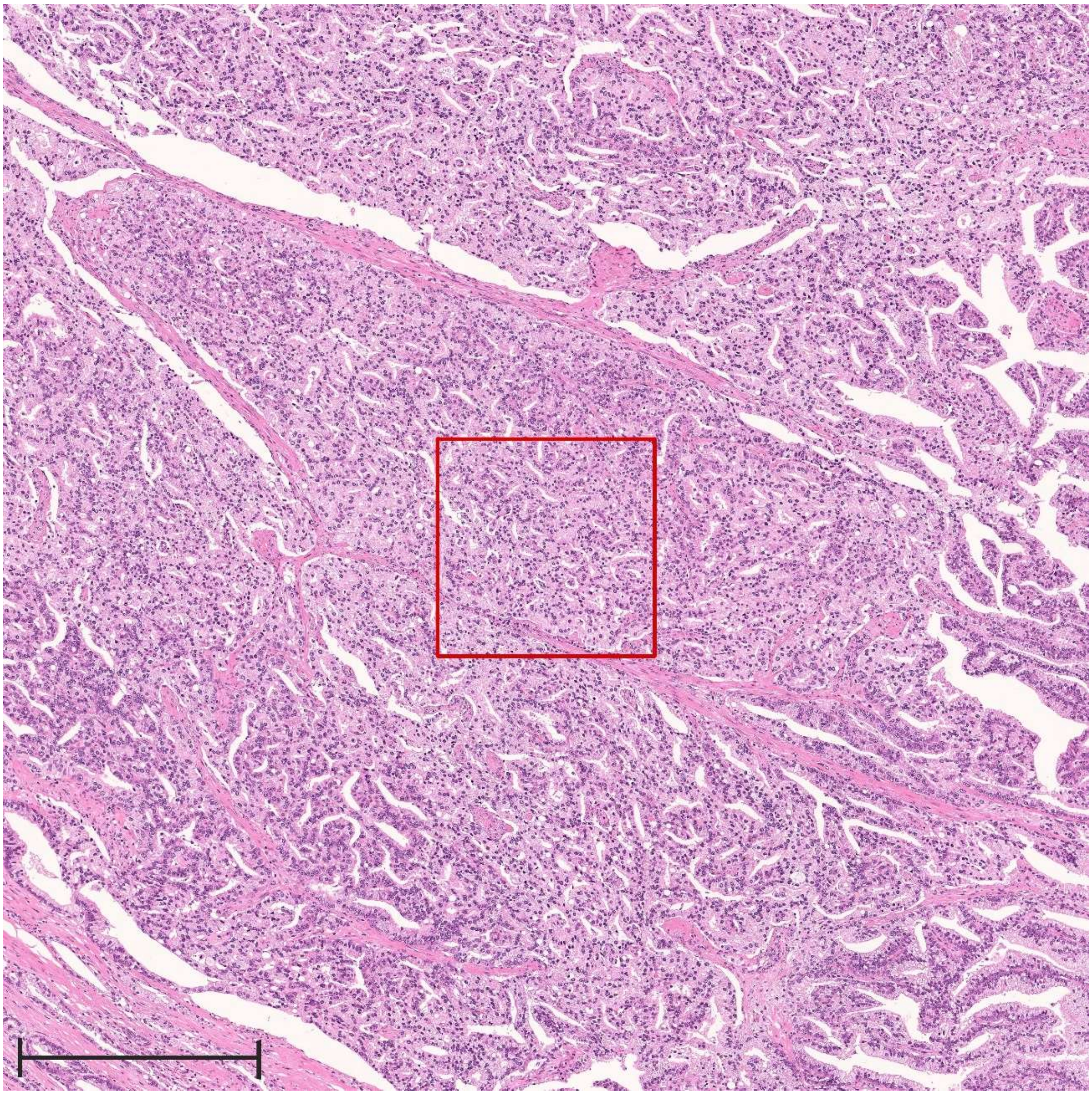

Supplementary Figure 8 | Representative image of a key feature (wide-range image).

Wide-range image contains a representative image from Figure 4b. The representative image was detected by our algorithm and is indicated with a red square. The scale bar included in the image represents a length of 500 μm. Expert genitourinary pathologist's comments: Adenocarcinoma. The area is entirely composed of adenocarcinoma cells with fused gland component, which is diagnosed as Gleason pattern 4.

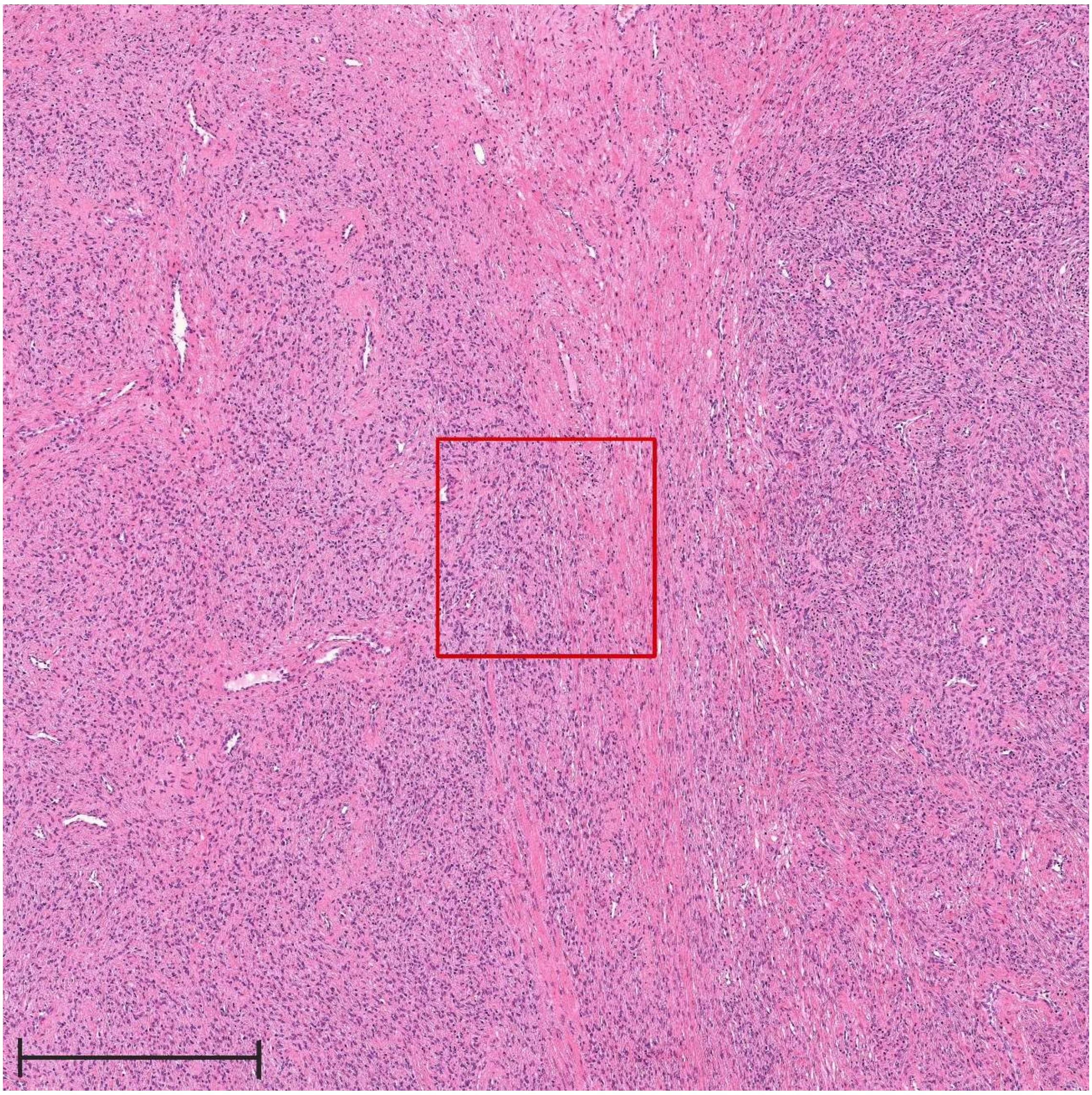

Supplementary Figure 9 | Representative image of a key feature (wide-range image).

Wide-range image contains a representative image from Figure 4c. The representative image was detected by our algorithm and is indicated with a red square. The scale bar included in the image represents a length of 500 µm. Expert genitourinary pathologist's comments: Stromal component without cancer cells tends to show dense cellularity.

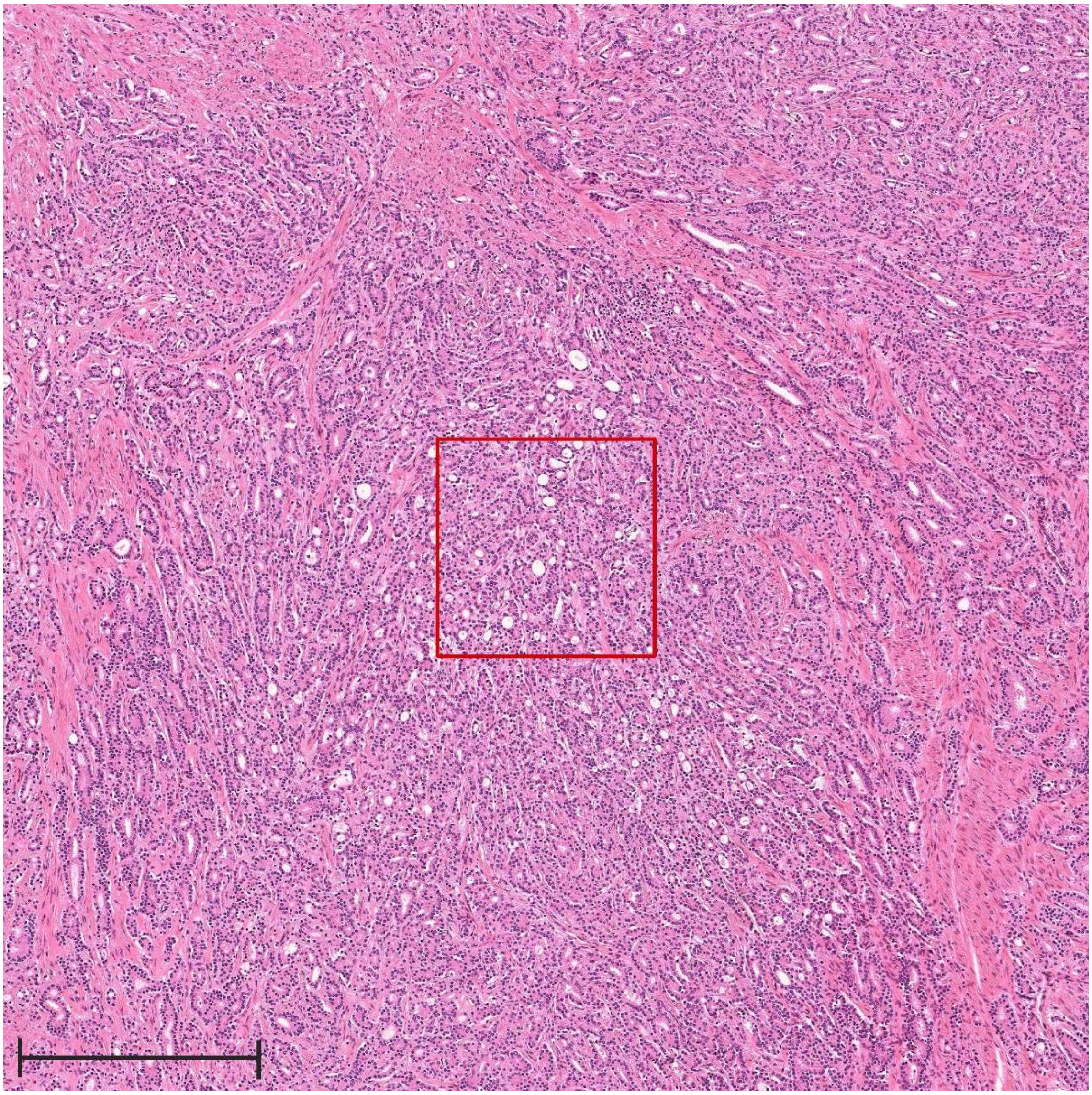

Supplementary Figure 10 | Representative image of a key feature (wide-range image).

Wide-range image contains a representative image from Figure 4d. The representative image was detected by our algorithm and is indicated with a red square. The scale bar included in the image represents a length of 500 μm. Expert genitourinary pathologist's comments: Adenocarcinoma. The area is entirely composed of adenocarcinoma cells with ill-defined gland formation, which is diagnosed as Gleason pattern 4.

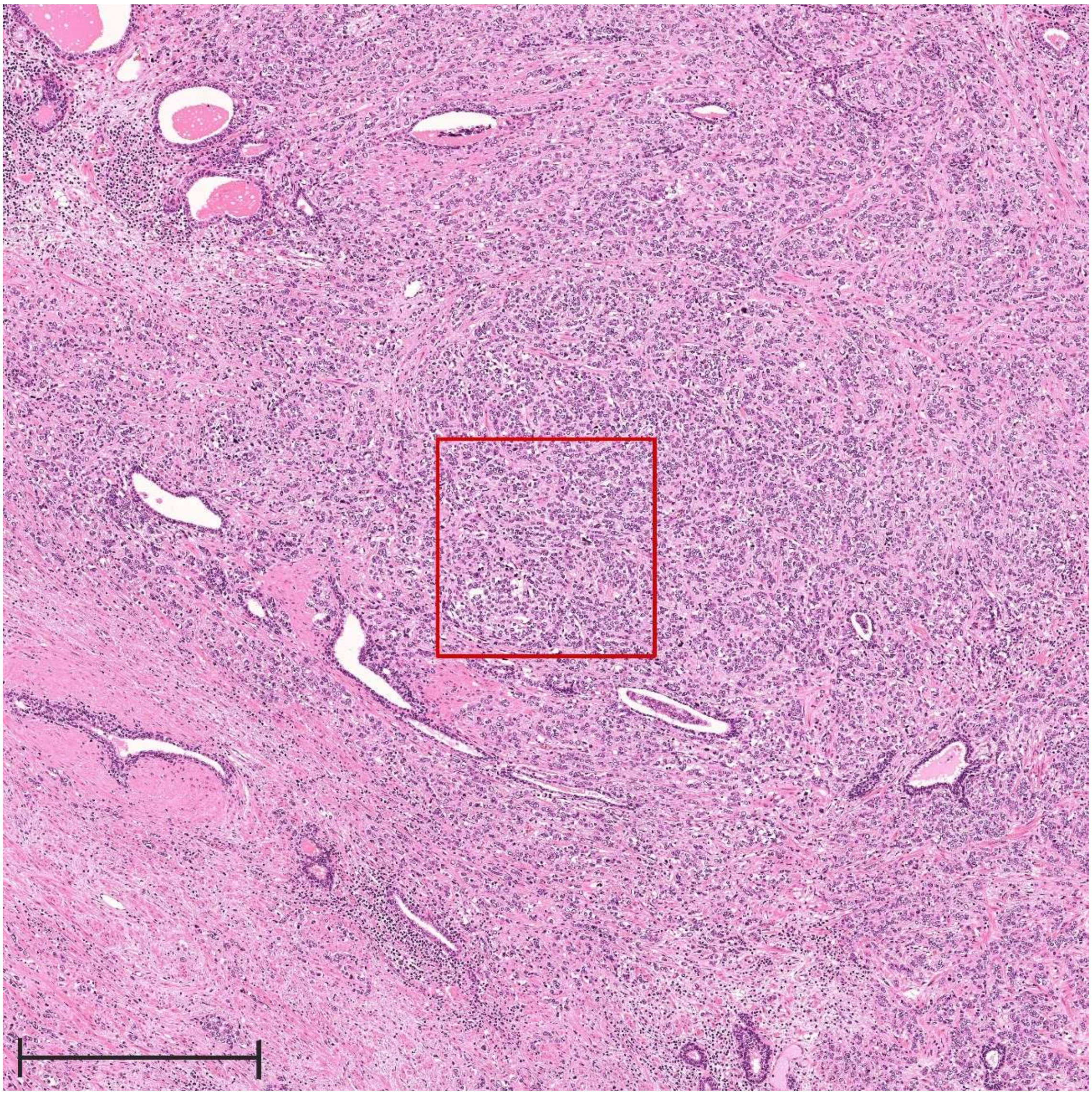

Supplementary Figure 11 | Representative image of a key feature (wide-range image).

Wide-range image contains a representative image from Figure 4e. The representative image was detected by our algorithm and is indicated with a red square. The scale bar included in the image represents a length of 500 µm. Expert genitourinary pathologist’s comments: Adenocarcinoma. The red squared area is entirely composed of adenocarcinoma cells without gland formation, which is diagnosed as Gleason pattern 5. Other area is also mostly composed of Gleason pattern 5 component.

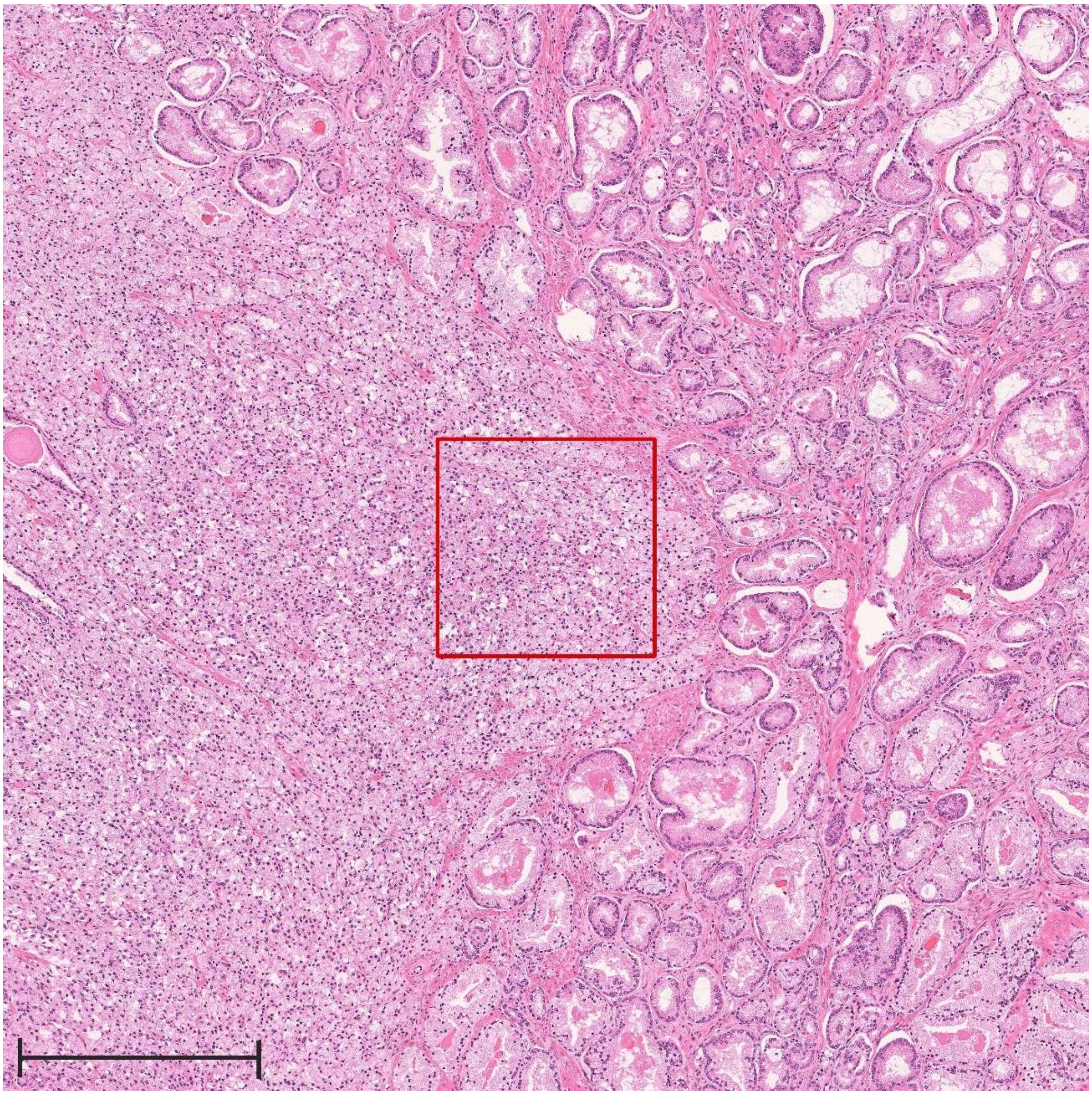

Supplementary Figure 12 | Representative image of a key feature (wide-range image).

Wide-range image contains a representative image from Figure 4f. The representative image was detected by our algorithm and is indicated with a red square. The scale bar included in the image represents a length of 500 µm. Expert genitourinary pathologist's comments: Adenocarcinoma. The red squared area is entirely composed of adenocarcinoma cells without gland formation, which is diagnosed as Gleason pattern 5. Gleason pattern 3 component is also present in the right side.

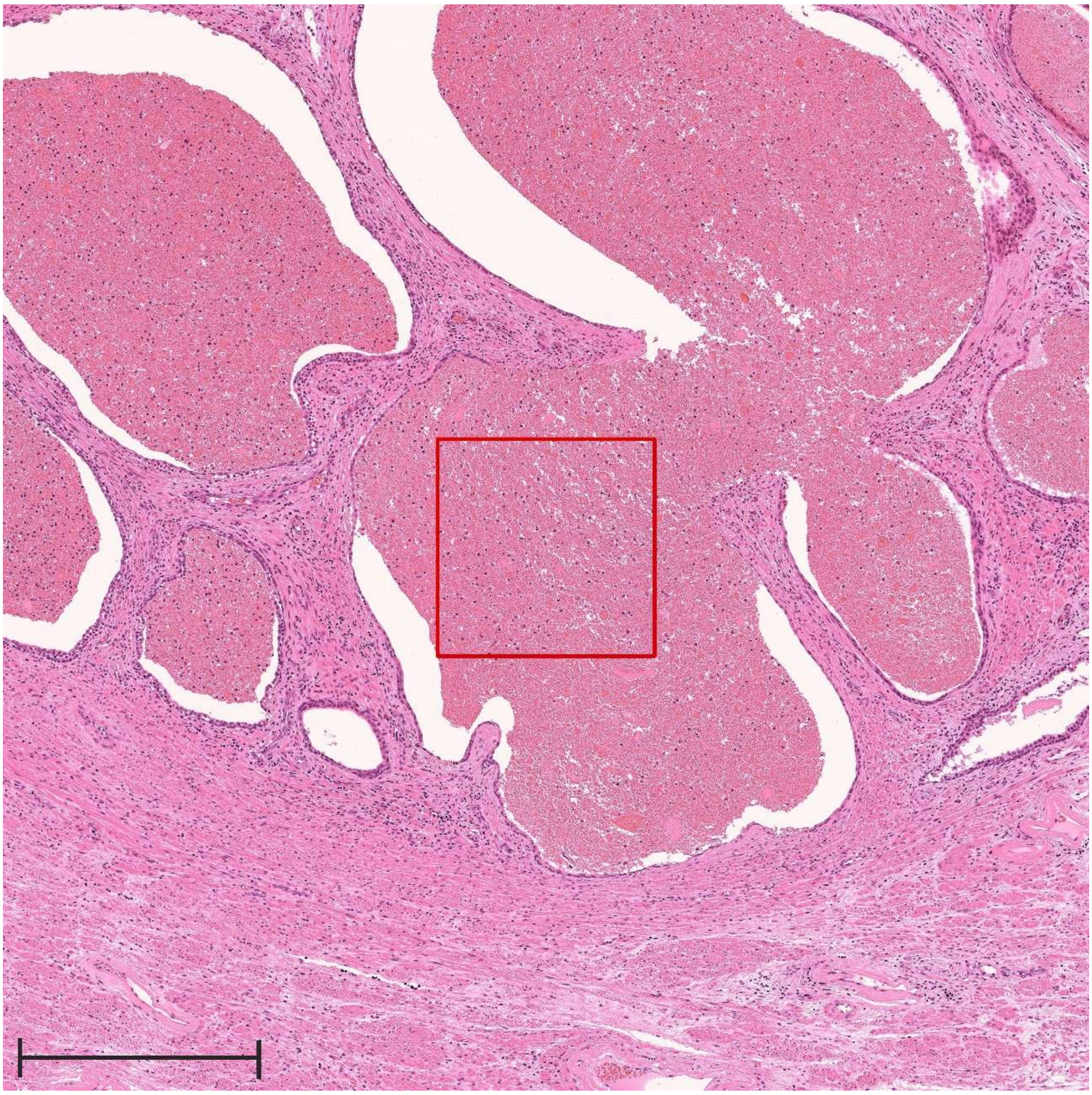

Supplementary Figure 13 | Representative image of a key feature (wide-range image).

Wide-range image contains a representative image from Figure 4g. The representative image was detected by our algorithm and is indicated with a red square. The scale bar included in the image represents a length of 500 µm. Expert genitourinary pathologist's comments: Dilated normal acinar glands with hemorrhage.

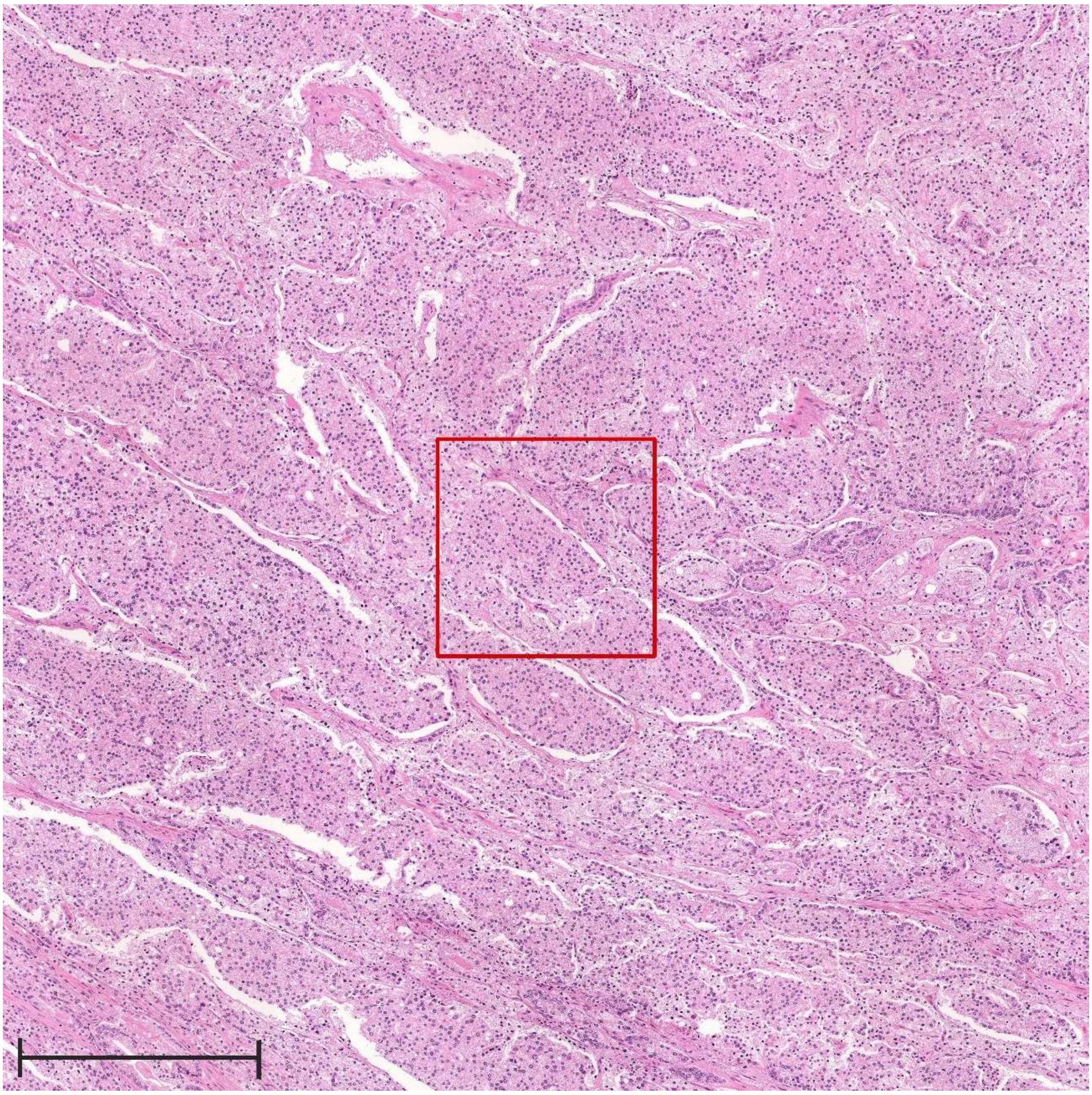

Supplementary Figure 14 | Representative image of a key feature (wide-range image).

Wide-range image contains a representative image from Figure 4h. The representative image was detected by our algorithm and is indicated with a red square. The scale bar included in the image represents a length of 500 µm. Expert genitourinary pathologist's comments: Adenocarcinoma. The red squared area is entirely composed of adenocarcinoma cells without gland formation, which is diagnosed as Gleason pattern 5. Gleason pattern 4 component showing fused glands is also present outside of the red square.

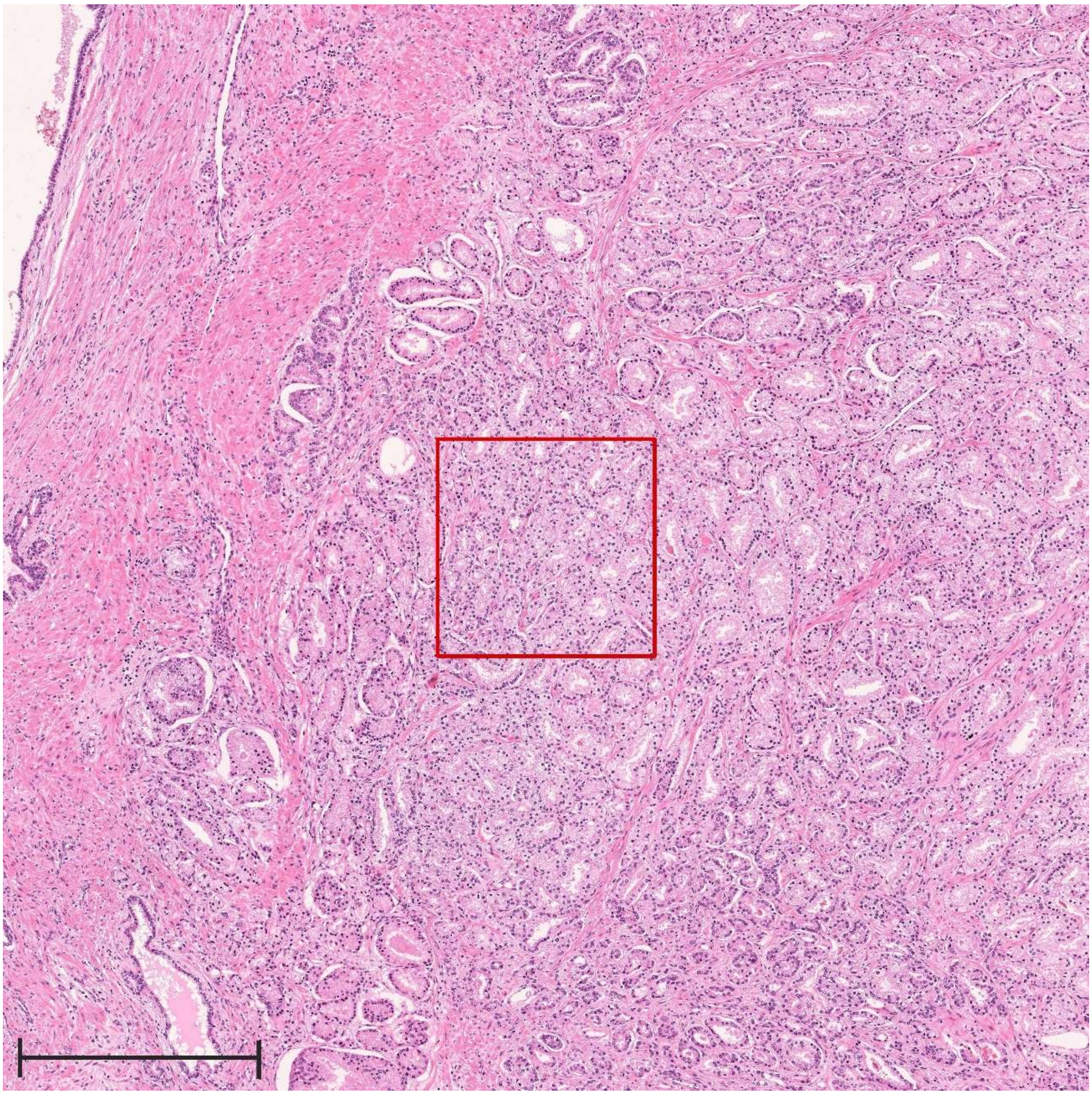

i

Supplementary Figure 15 | Representative image of a key feature (wide-range image).

Wide-range image contains a representative image from Figure 4i. The representative image was detected by our algorithm and is indicated with a red square. The scale bar included in the image represents a length of 500 µm. Expert genitourinary pathologist's comments: Adenocarcinoma. The red squared area is entirely composed of adenocarcinoma cells with well-formed glands. The tumor in the red square is predominantly occupied by Gleason pattern 4 component. Other area is also mostly composed of Gleason pattern 3 component.

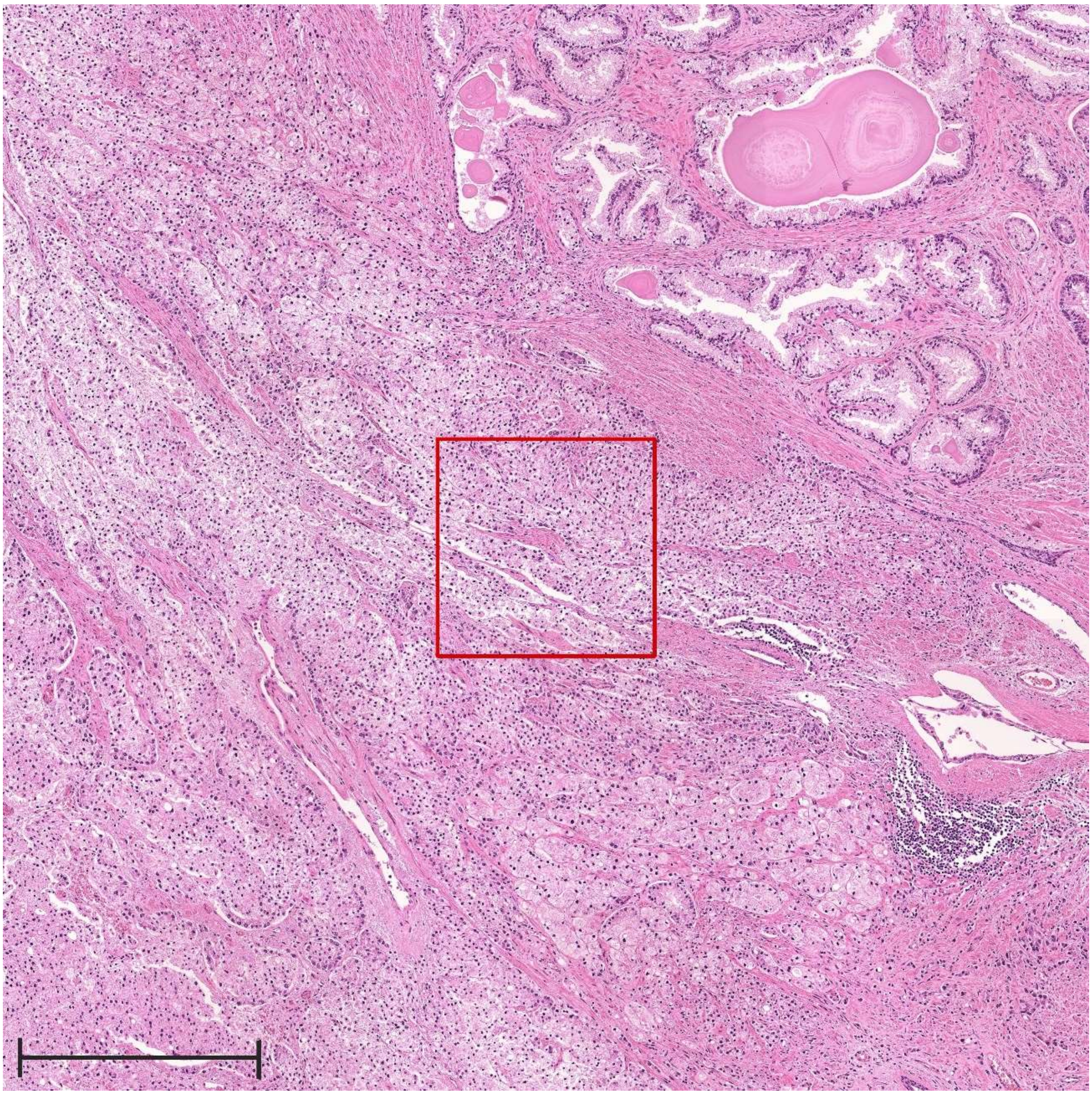

Supplementary Figure 16 | Representative image of a key feature (wide-range image).

Wide-range image contains a representative image from Figure 4j. The representative image was detected by our algorithm and is indicated with a red square. The scale bar included in the image represents a length of 500 μm. Expert genitourinary pathologist's comments: Adenocarcinoma. The red squared area is entirely composed of adenocarcinoma cells without gland formation, which is diagnosed as Gleason pattern 5. Other area is also mostly composed of Gleason pattern 5 component.

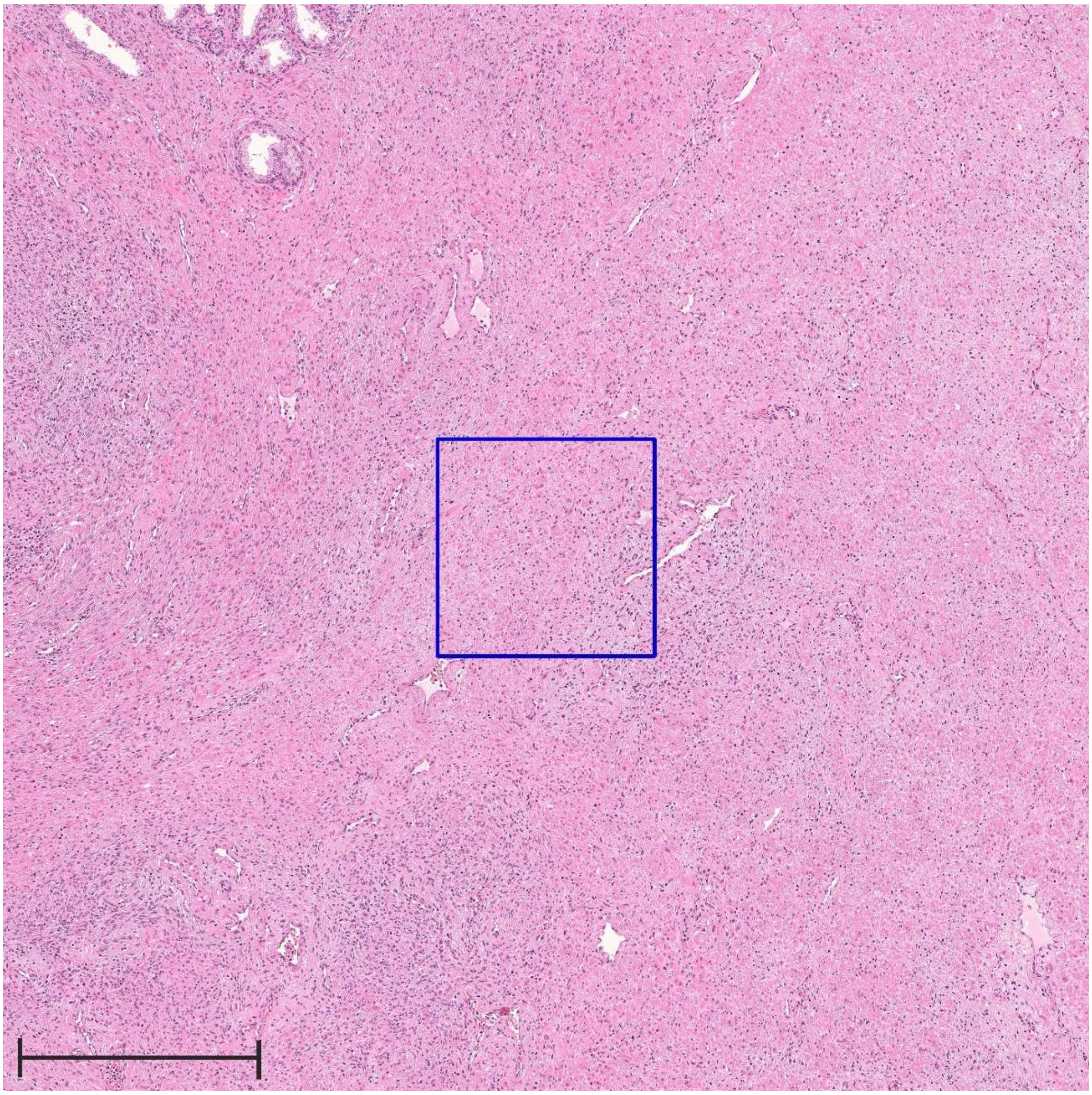

Supplementary Figure 17 | Representative image of a key feature (wide-range image).

Wide-range image contains a representative image from Figure 4k. The representative image was detected by our algorithm and is indicated with a blue square. The scale bar included in the image represents a length of 500 µm. Expert genitourinary pathologist's comments: Almost normal fibrous tissue at the transitional zone in the prostate.

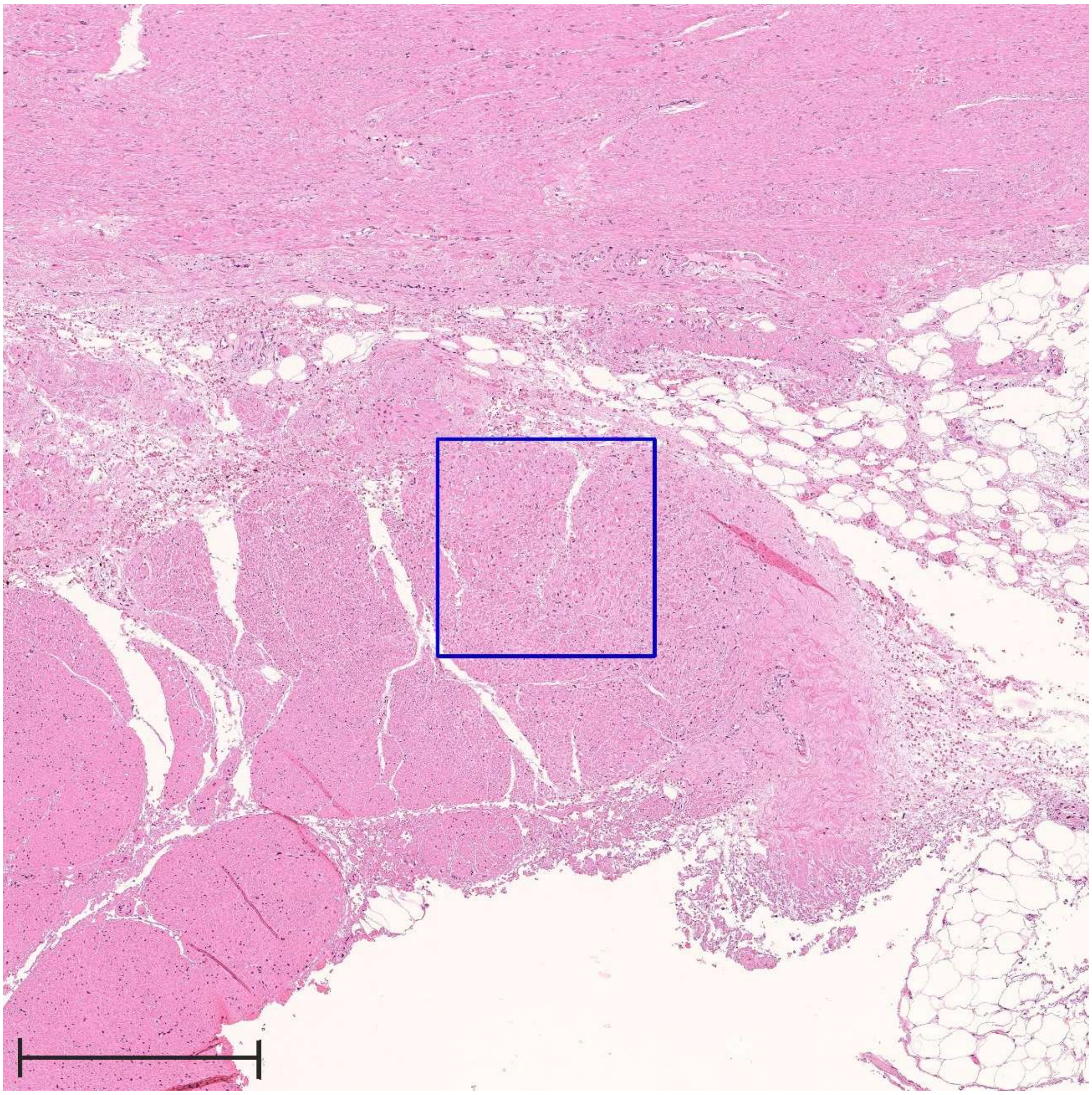

Supplementary Figure 18 | Representative image of a key feature (wide-range image).

Wide-range image contains a representative image from Figure 4l. The representative image was detected by our algorithm and is indicated with a blue square. The scale bar included in the image represents a length of 500 μm. Expert genitourinary pathologist's comments: Almost normal fibromuscular tissue at the extraprostatic area.

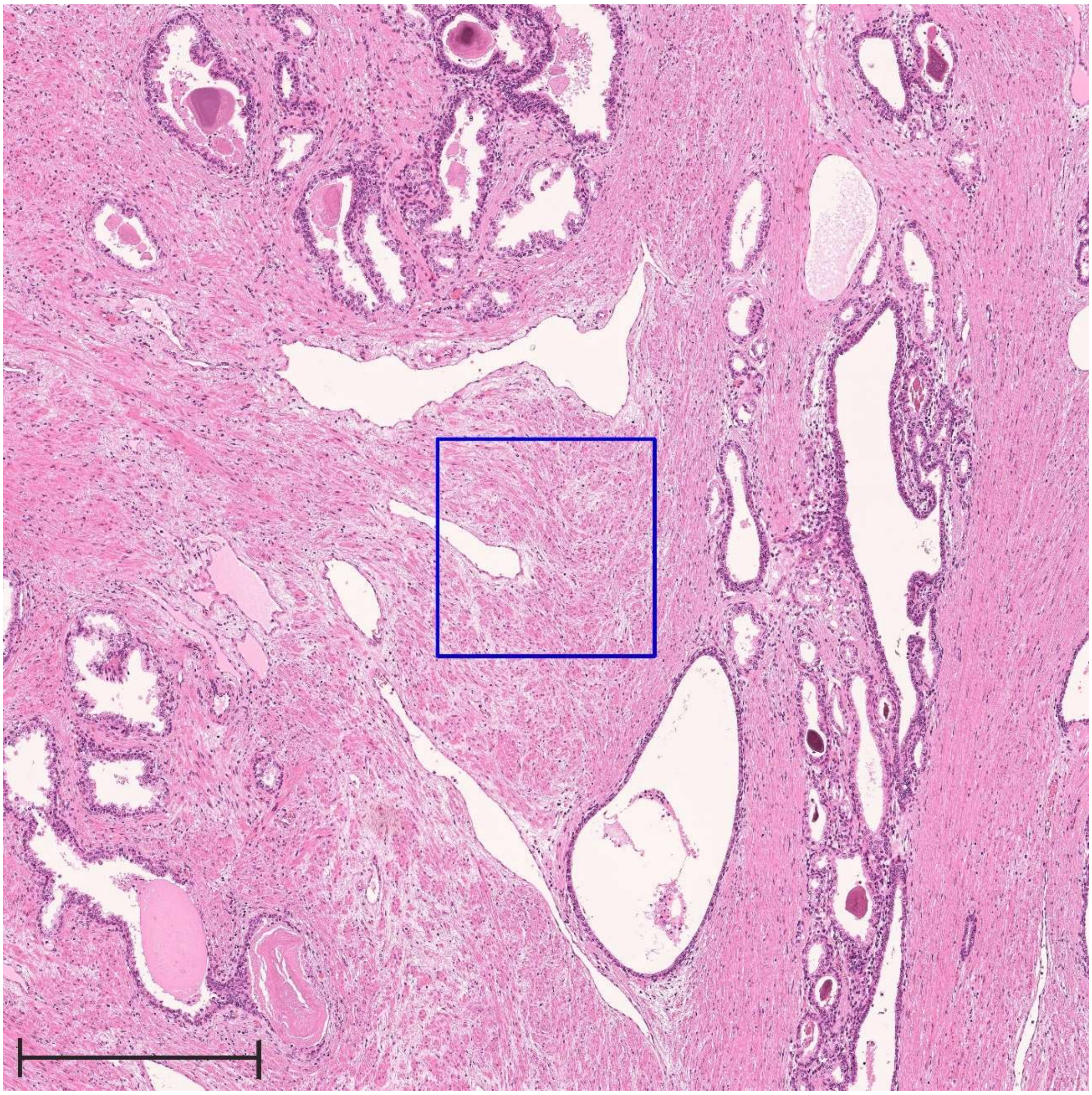

Supplementary Figure 19 | Representative image of a key feature (wide-range image).

Wide-range image contains a representative image from Figure 4m. The representative image was detected by our algorithm and is indicated with a blue square. The scale bar included in the image represents a length of 500 μm. Expert genitourinary pathologist’s comments: Almost normal smooth muscle tissue at the peripheral zone in the prostate.

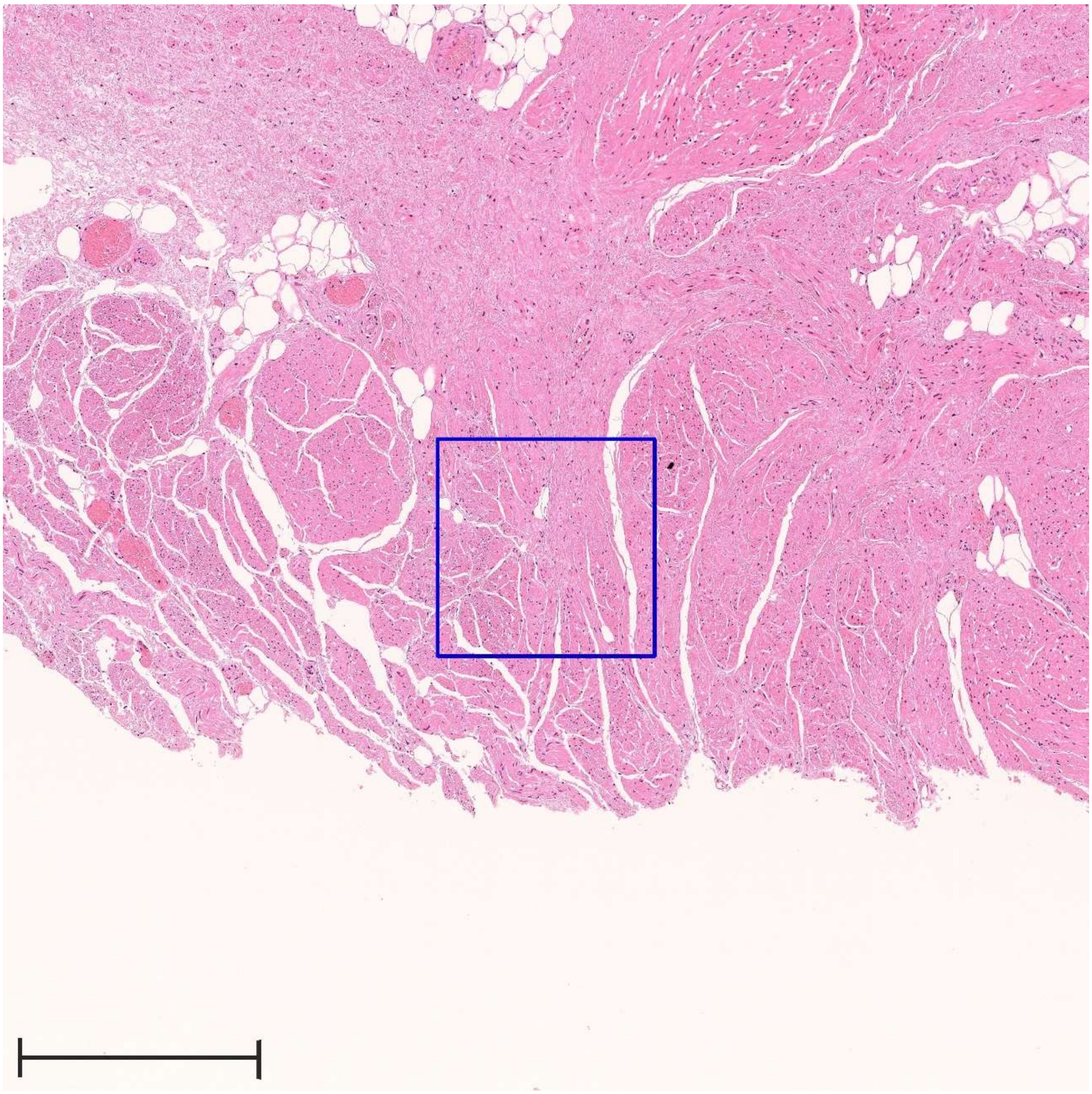

Supplementary Figure 20 | Representative image of a key feature (wide-range image).

Wide-range image contains a representative image from Figure 4n. The representative image was detected by our algorithm and is indicated with a blue square. The scale bar included in the image represents a length of 500 μm. Expert genitourinary pathologist's comments: Almost normal fibromuscular tissue at the extraprostatic area.

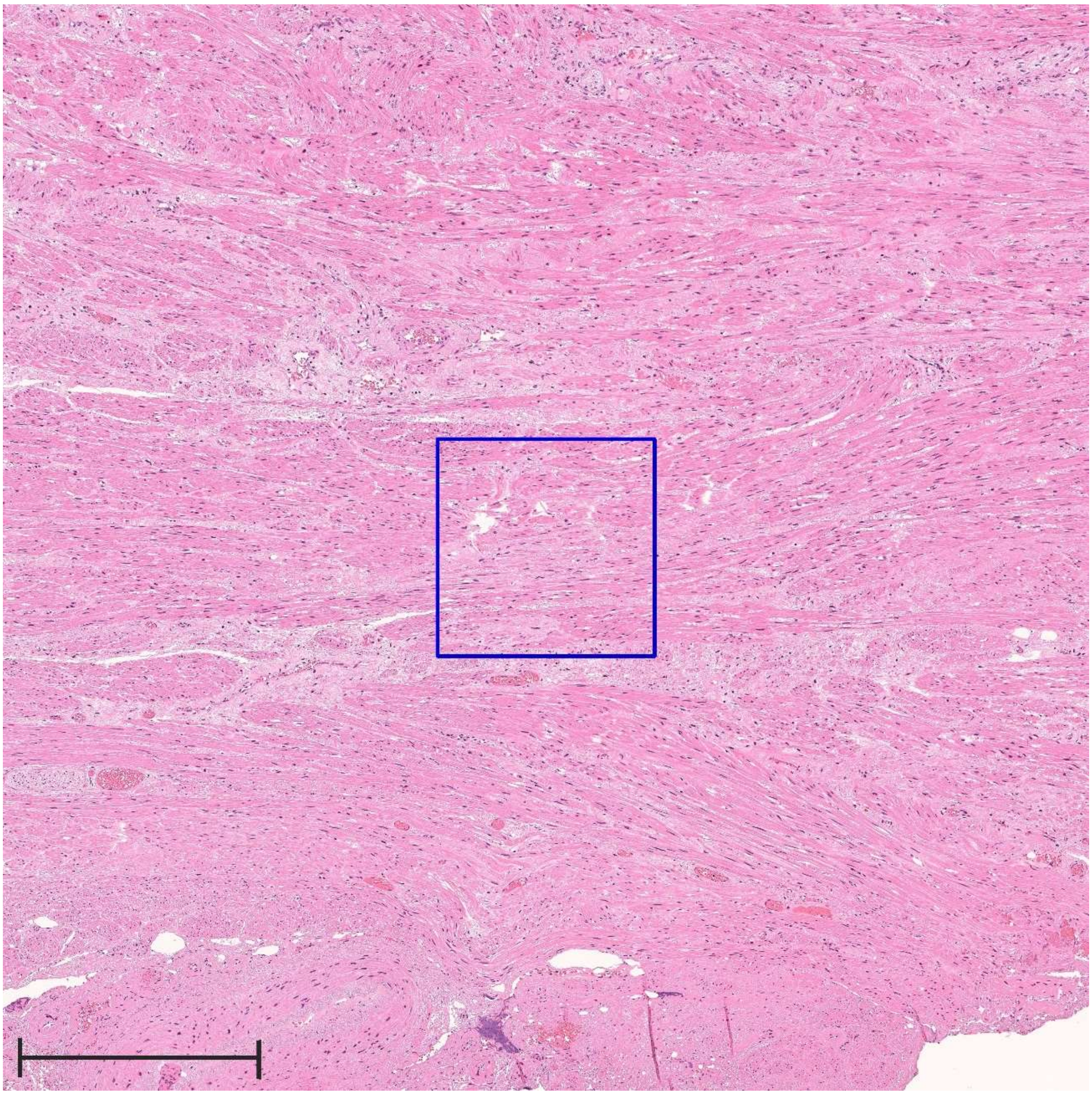

Supplementary Figure 21 | Representative image of a key feature (wide-range image).

Wide-range image contains a representative image from Figure 4o. The representative image was detected by our algorithm and is indicated with a blue square. The scale bar included in the image represents a length of 500 μm. Expert genitourinary pathologist's comments: Almost normal smooth muscle tissue at the transitional zone in the prostate.

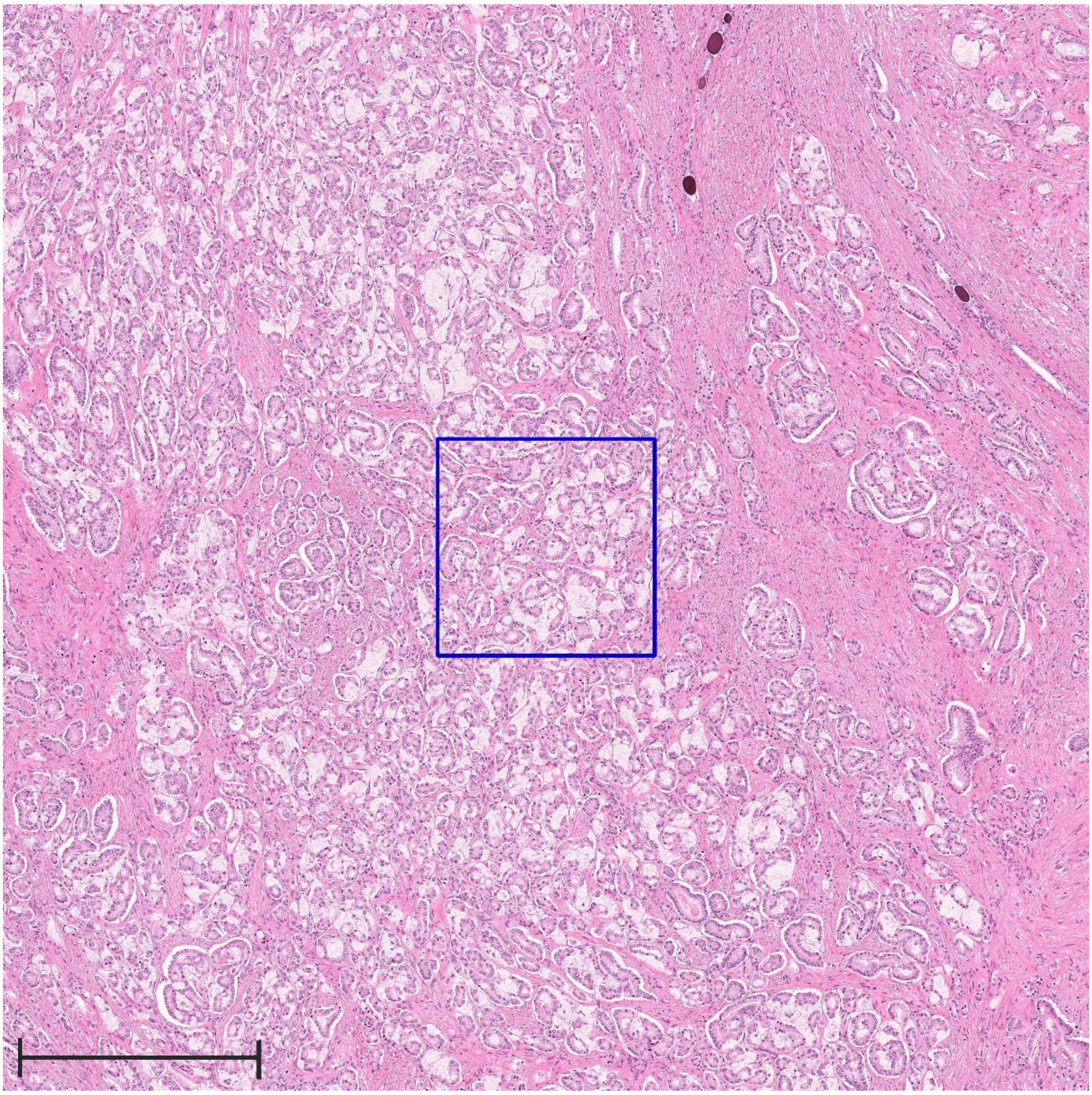

Supplementary Figure 22 | Representative image of a key feature (wide-range image).

Wide-range image contains a representative image from Figure 4p. The representative image was detected by our algorithm and is indicated with a blue square. The scale bar included in the image represents a length of 500 µm. Expert genitourinary pathologist's comments: Adenocarcinoma with focal mucin extravasation. The cancer area is mostly composed of Gleason pattern 3.

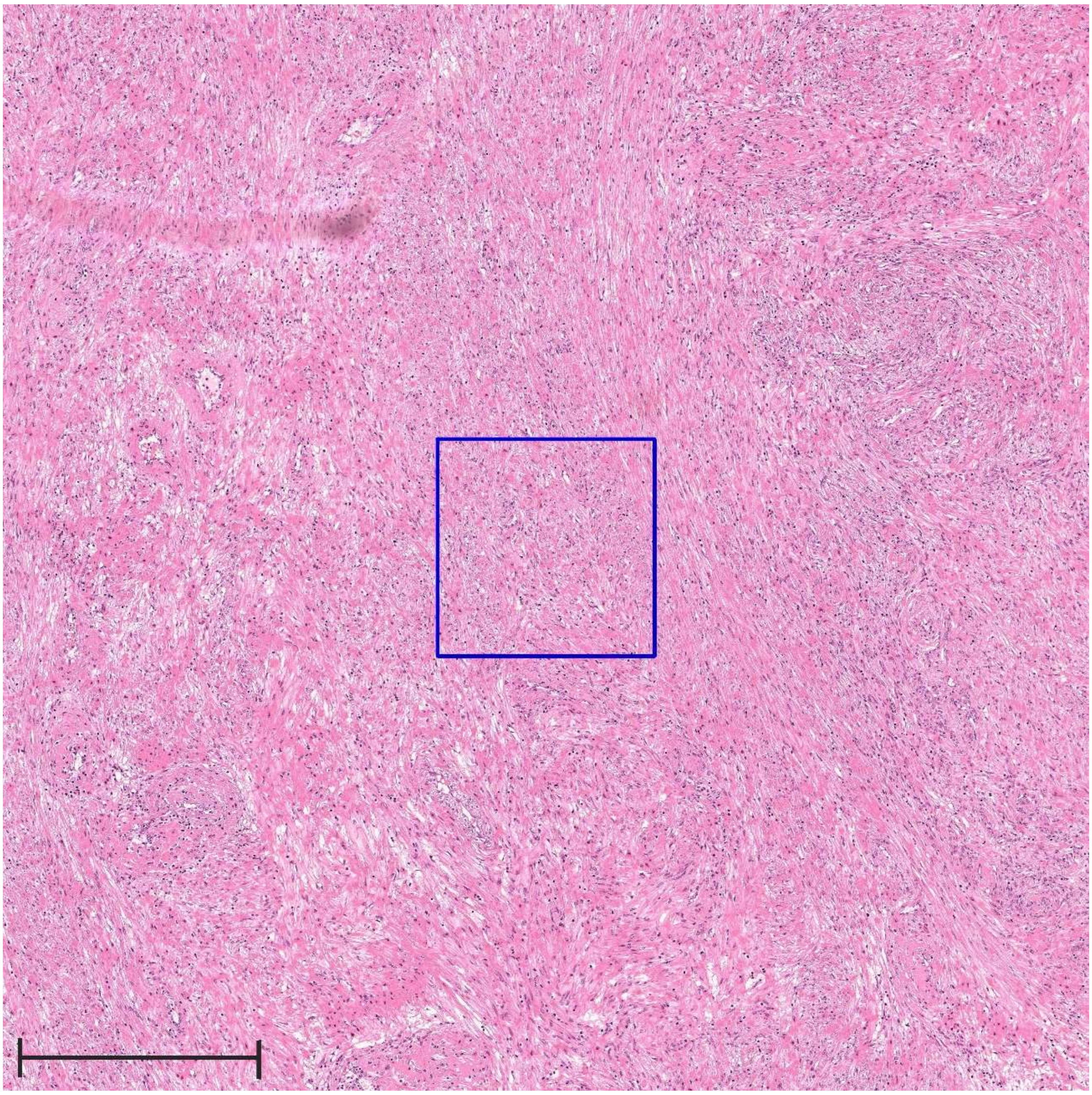

Supplementary Figure 23 | Representative image of a key feature (wide-range image).

Wide-range image contains a representative image from Figure 4q. The representative image was detected by our algorithm and is indicated with a blue square. The scale bar included in the image represents a length of 500 μm. Expert genitourinary pathologist's comments: Almost normal smooth muscle tissue in the prostate. The location cannot be determined.

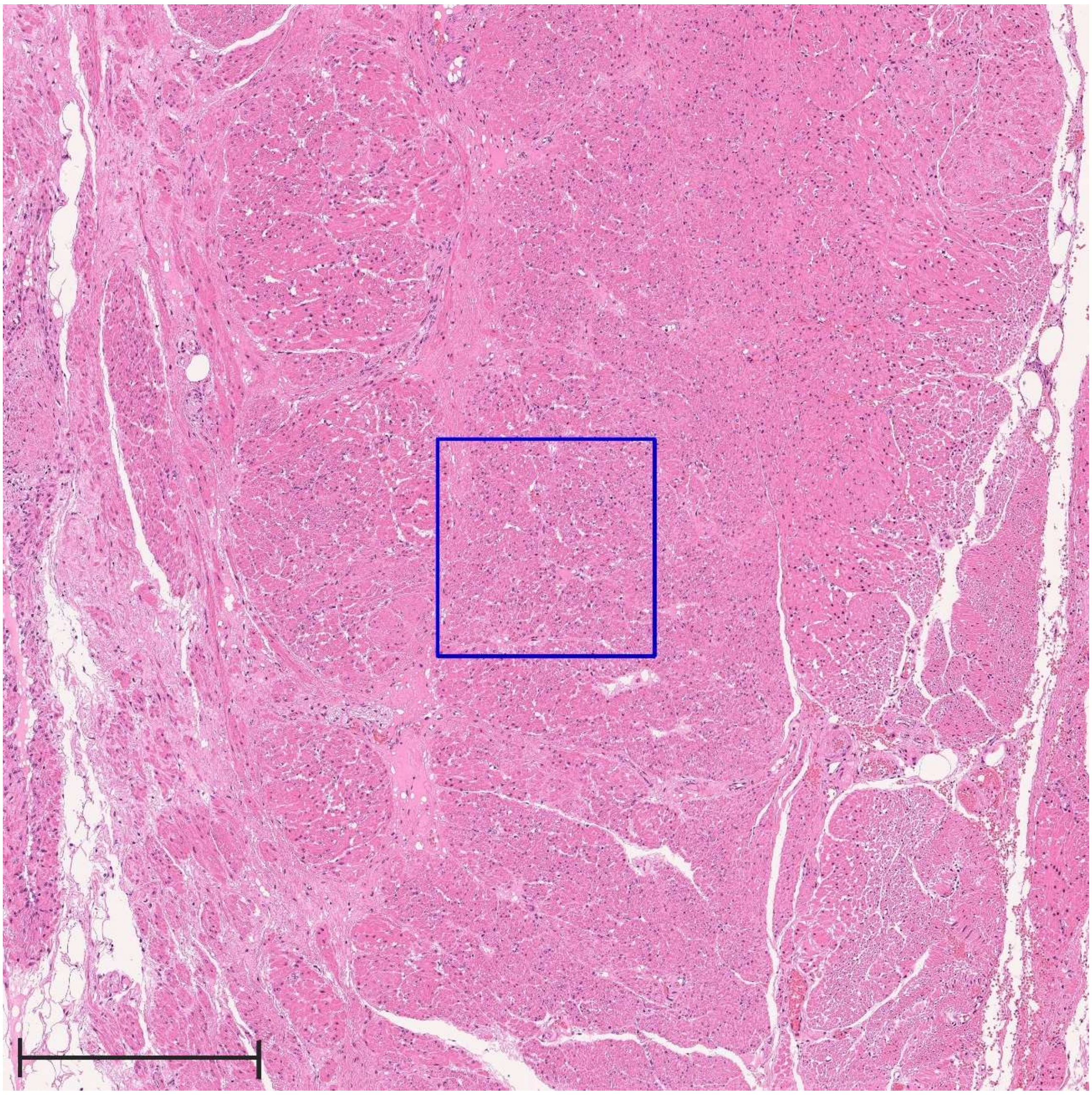

Supplementary Figure 24 | Representative image of a key feature (wide-range image).

Wide-range image contains a representative image from Figure 4r. The representative image was detected by our algorithm and is indicated with a blue square. The scale bar included in the image represents a length of 500 µm. Expert genitourinary pathologist's comments: Almost normal fibromuscular tissue at the extraprostatic area.

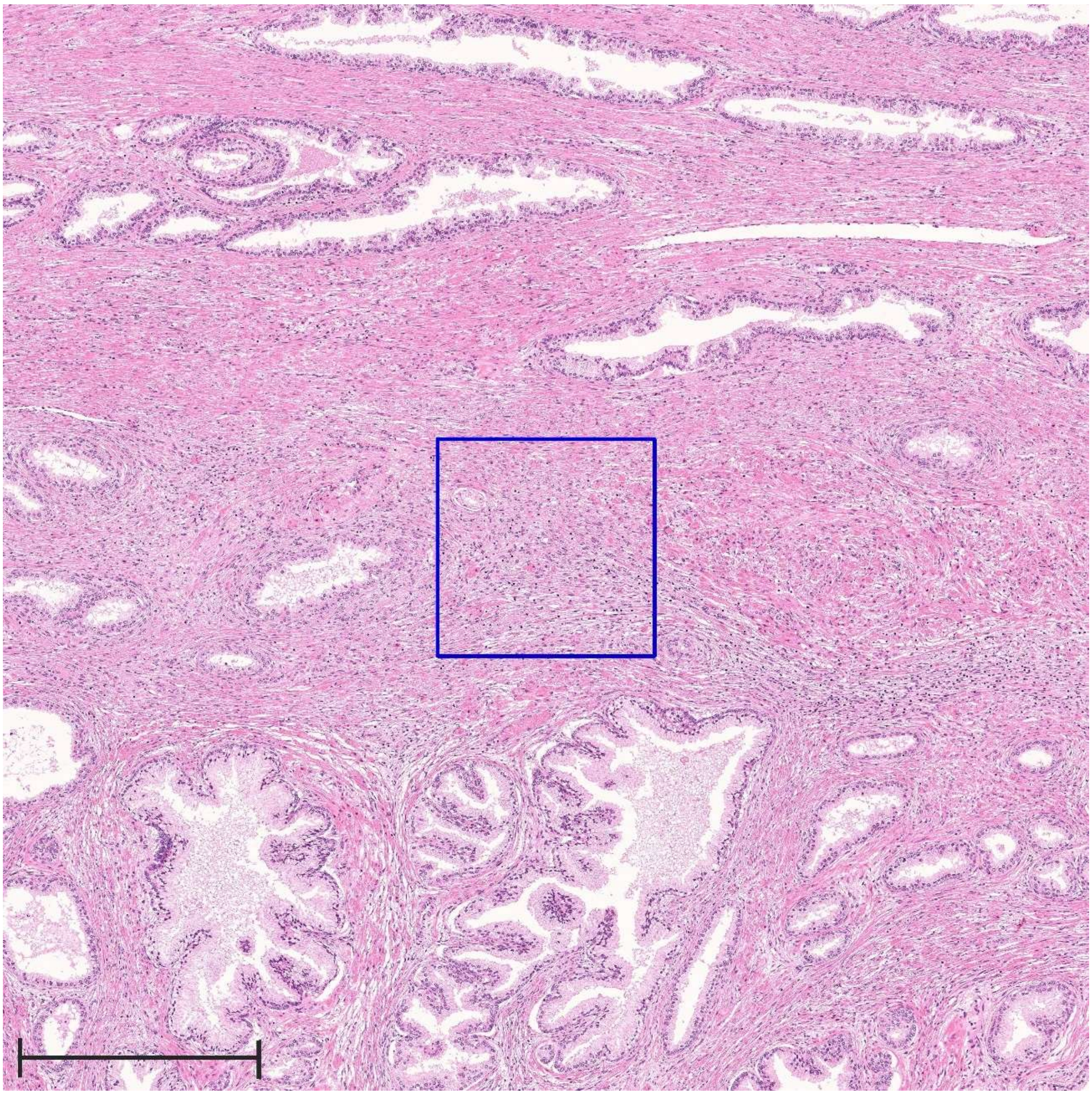

Supplementary Figure 25 | Representative image of a key feature (wide-range image).

Wide-range image contains a representative image from Figure 4s. The representative image was detected by our algorithm and is indicated with a blue square. The scale bar included in the image represents a length of 500 μm. Expert genitourinary pathologist's comments: Almost normal fibromuscular tissue at the peripheral zone in the prostate.

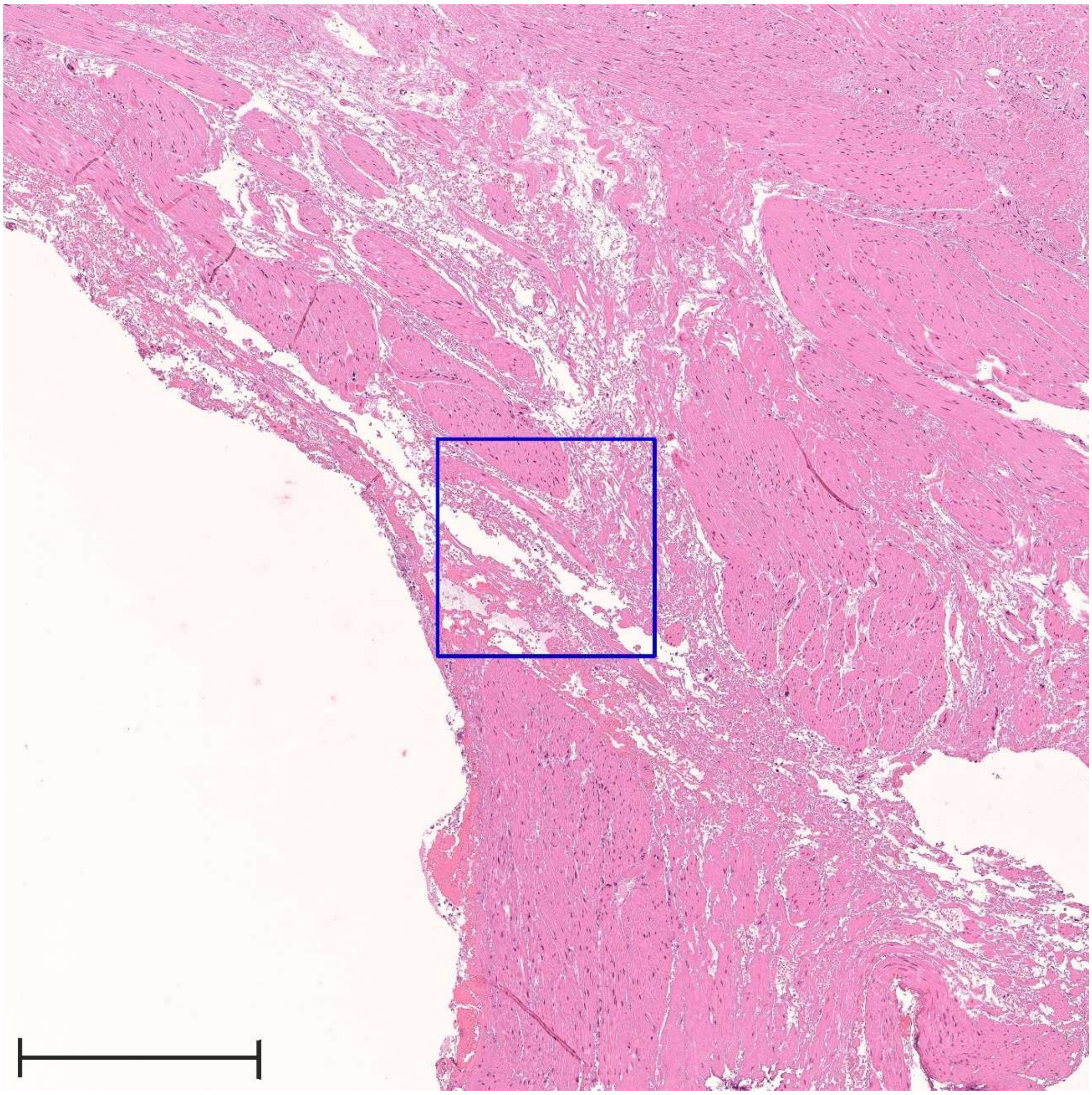

Supplementary Figure 26 | Representative image of a key feature (wide-range image).

Wide-range image contains a representative image from Figure 4t. The representative image was detected by our algorithm and is indicated with a blue square. The scale bar included in the image represents a length of 500 μm. Expert genitourinary pathologist's comments: Almost normal smooth muscle tissue at the surgical margin.

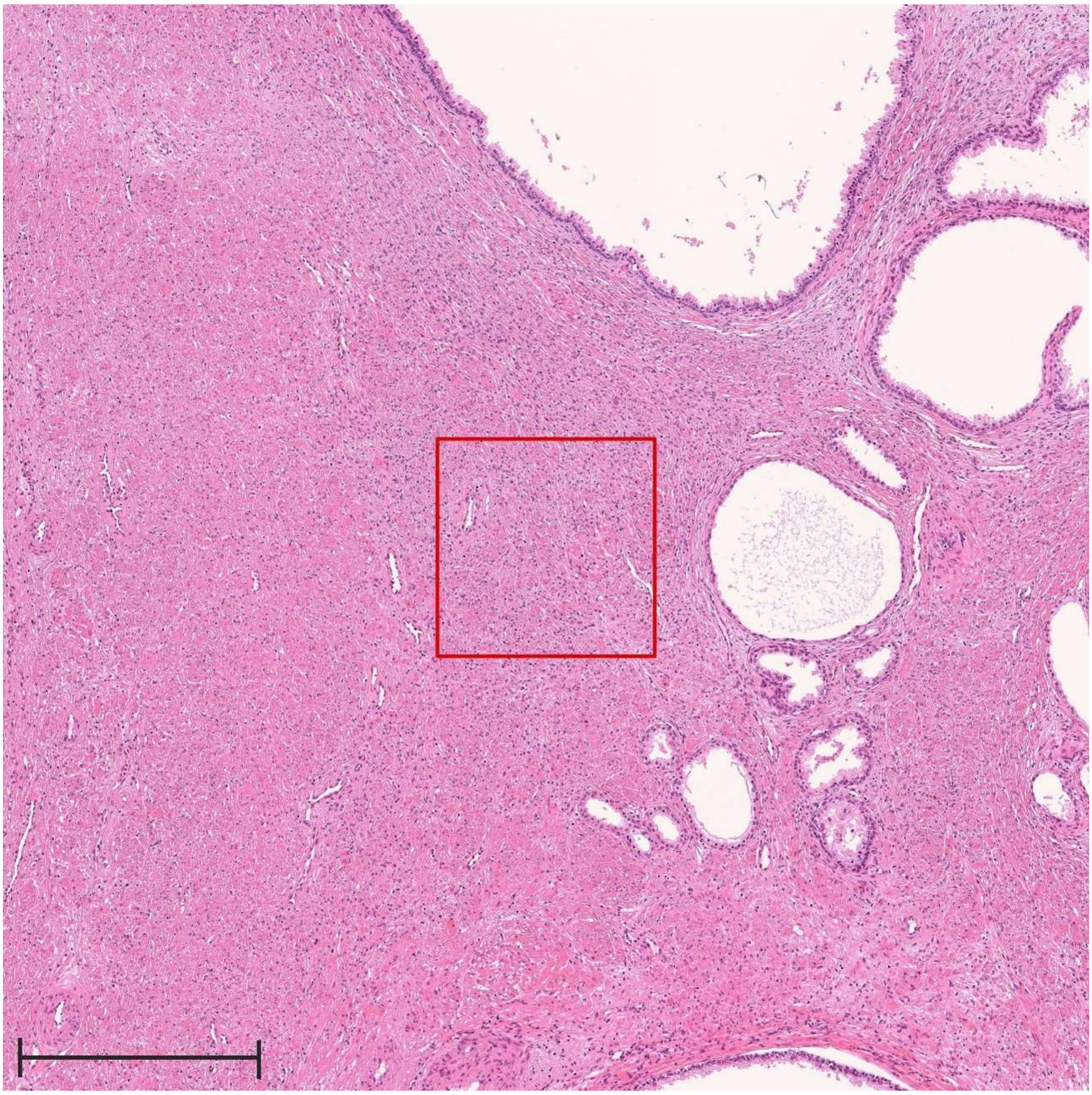

Supplementary Figure 27 | Representative image of a key feature (wide-range image).

Wide-range image contains a representative image of a weak predictor for biochemical recurrence. The representative image was detected by our algorithm and is indicated with a red square. The scale bar included in the image represents a length of 500 μm. Expert genitourinary pathologist's comments: Stromal component without cancer cells tends to show dense cellularity compared to those of normal structure. In addition, this figure includes dilated prostatic glands.

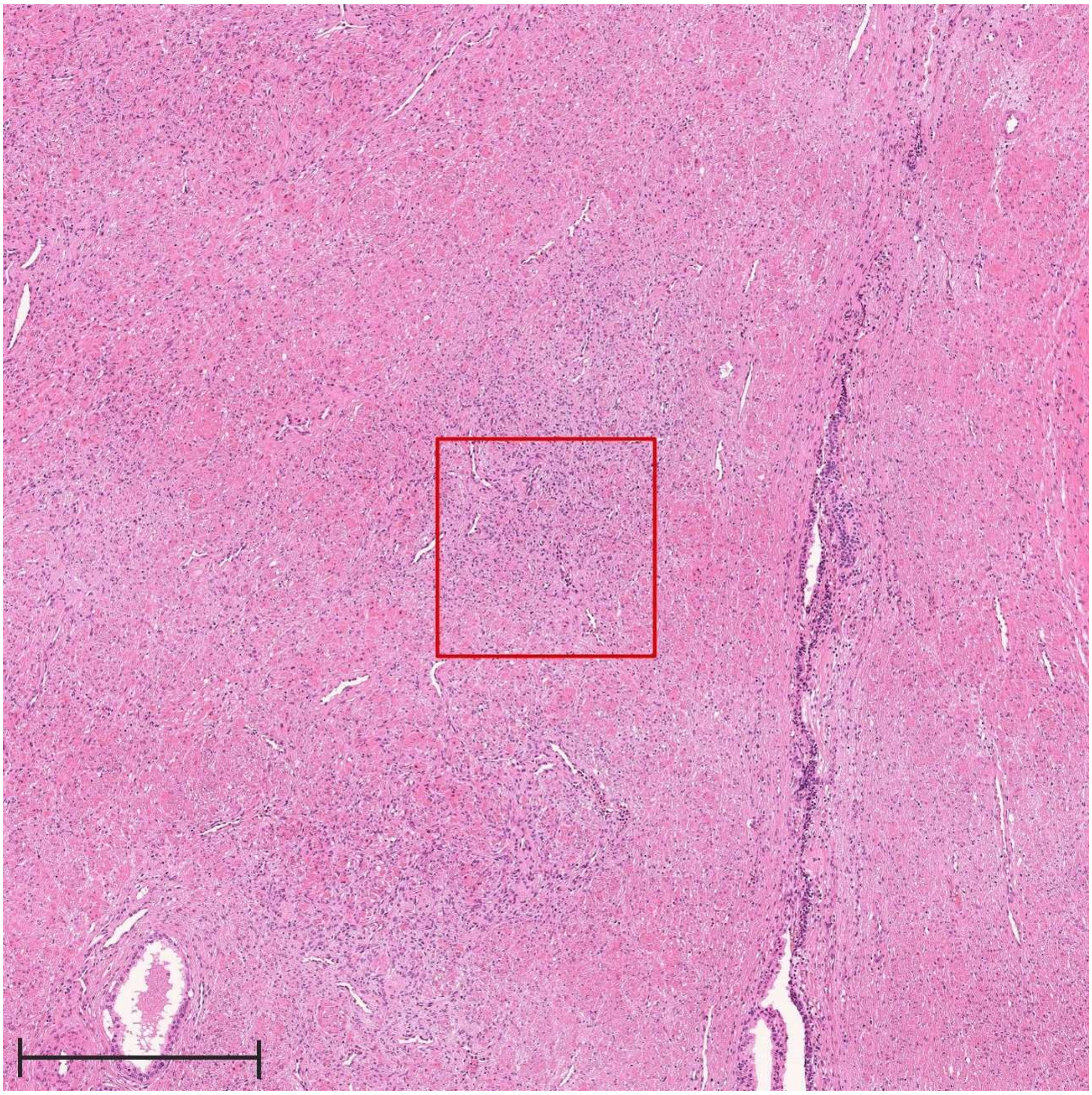

Supplementary Figure 28 | Representative image of a key feature (wide-range image).

Wide-range image contains a representative image of a weak predictor for biochemical recurrence. The representative image was detected by our algorithm and is indicated with a red square. The scale bar included in the image represents a length of 500 μm. Expert genitourinary pathologist's comments: Stromal component without cancer cells tends to show dense cellularity compared to those of normal structure.

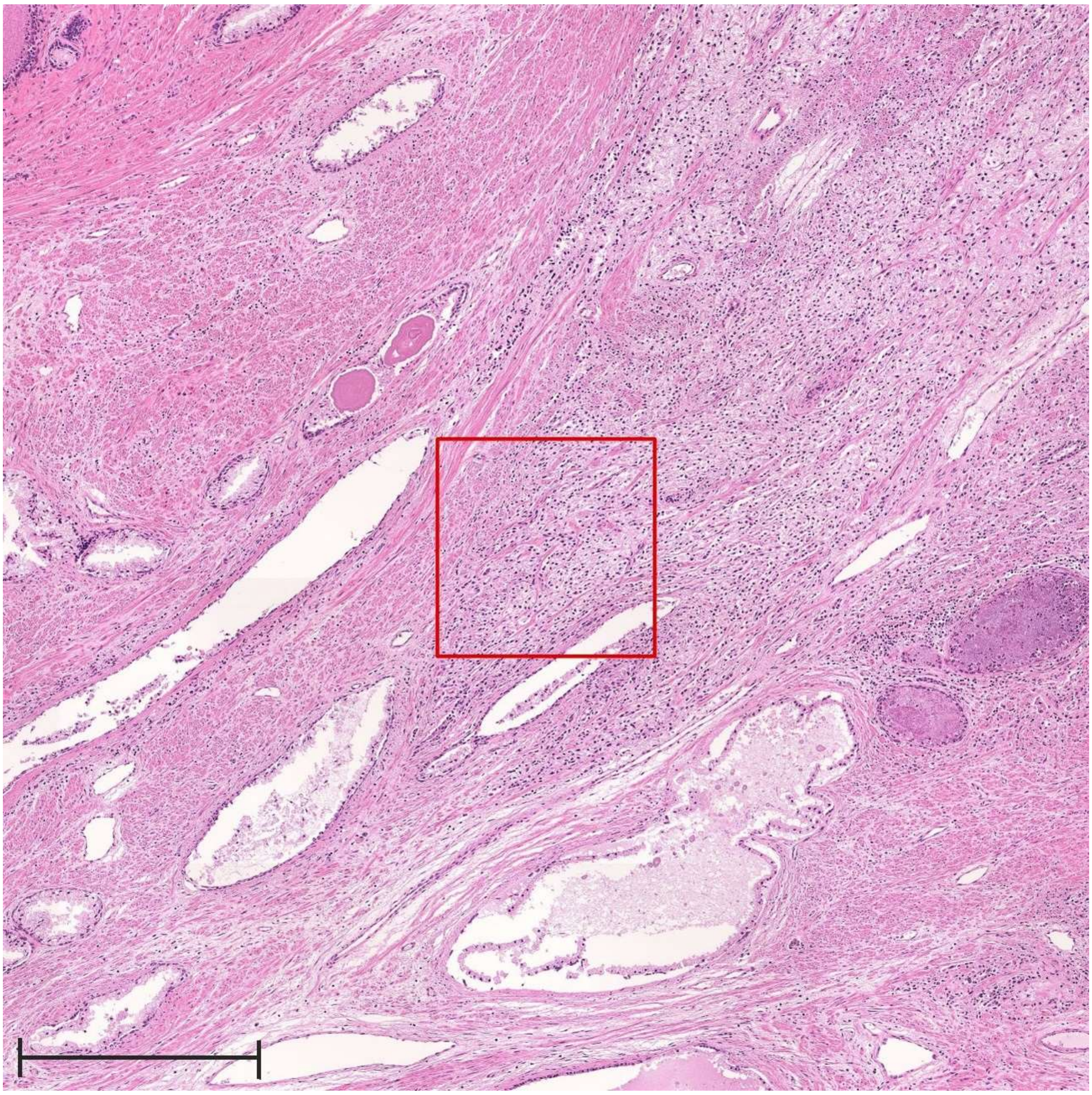

Supplementary Figure 29 | Representative image of a key feature (wide-range image).

Wide-range image contains a representative image of a weak predictor for biochemical recurrence. The representative image was detected by our algorithm and is indicated with a red square. The scale bar included in the image represents a length of 500 μm. Expert genitourinary pathologist's comments: Adenocarcinoma. The red squared shows border area between normal tissue and adenocarcinoma, which is diagnosed as Gleason pattern 5.

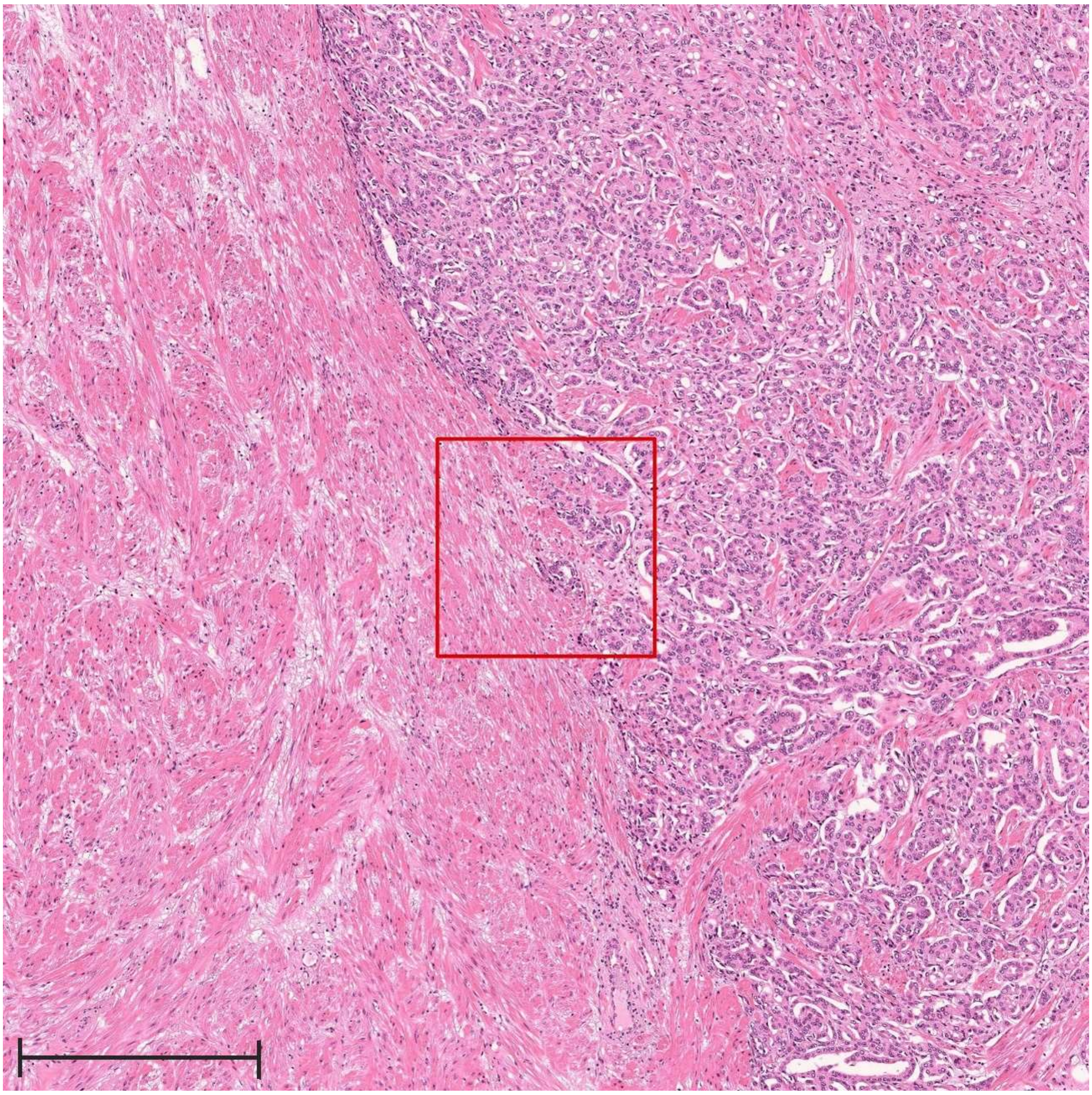

Supplementary Figure 30 | Representative image of a key feature (wide-range image).

Wide-range image contains a representative image of a weak predictor for biochemical recurrence. The representative image was detected by our algorithm and is indicated with a red square. The scale bar included in the image represents a length of 500 μm. Expert genitourinary pathologist's comments: Adenocarcinoma. The red squared shows border area between normal tissue and adenocarcinoma, which is diagnosed as Gleason pattern 4.

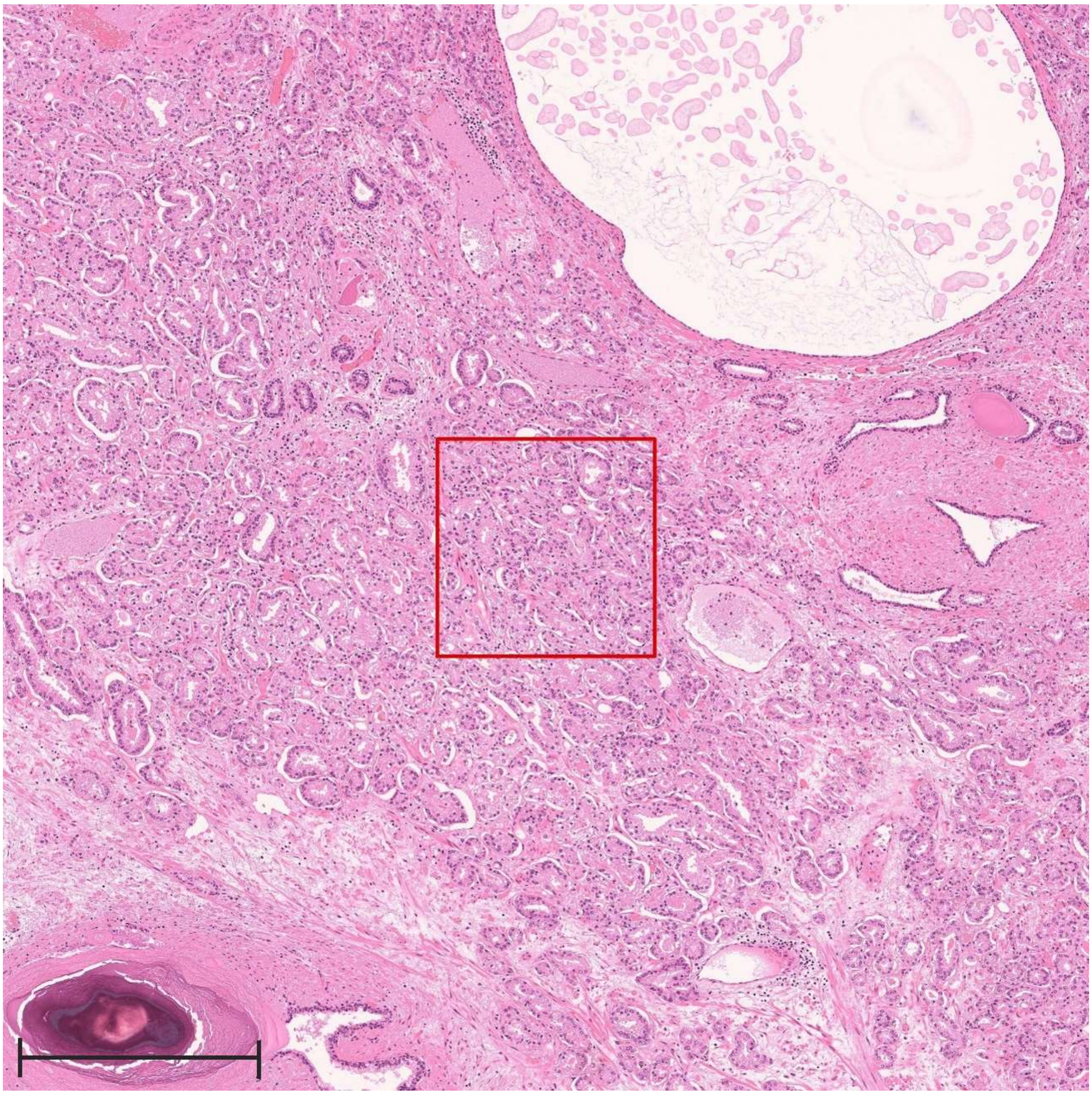

Supplementary Figure 31 | Representative image of a key feature (wide-range image).

Wide-range image contains a representative image of a weak predictor for biochemical recurrence. The representative image was detected by our algorithm and is indicated with a red square. The scale bar included in the image represents a length of 500 µm. Expert genitourinary pathologist's comments: Adenocarcinoma. The area shows adenocarcinoma cells, which is predominantly composed of Gleason pattern 3 (subsequently Gleason pattern 4).

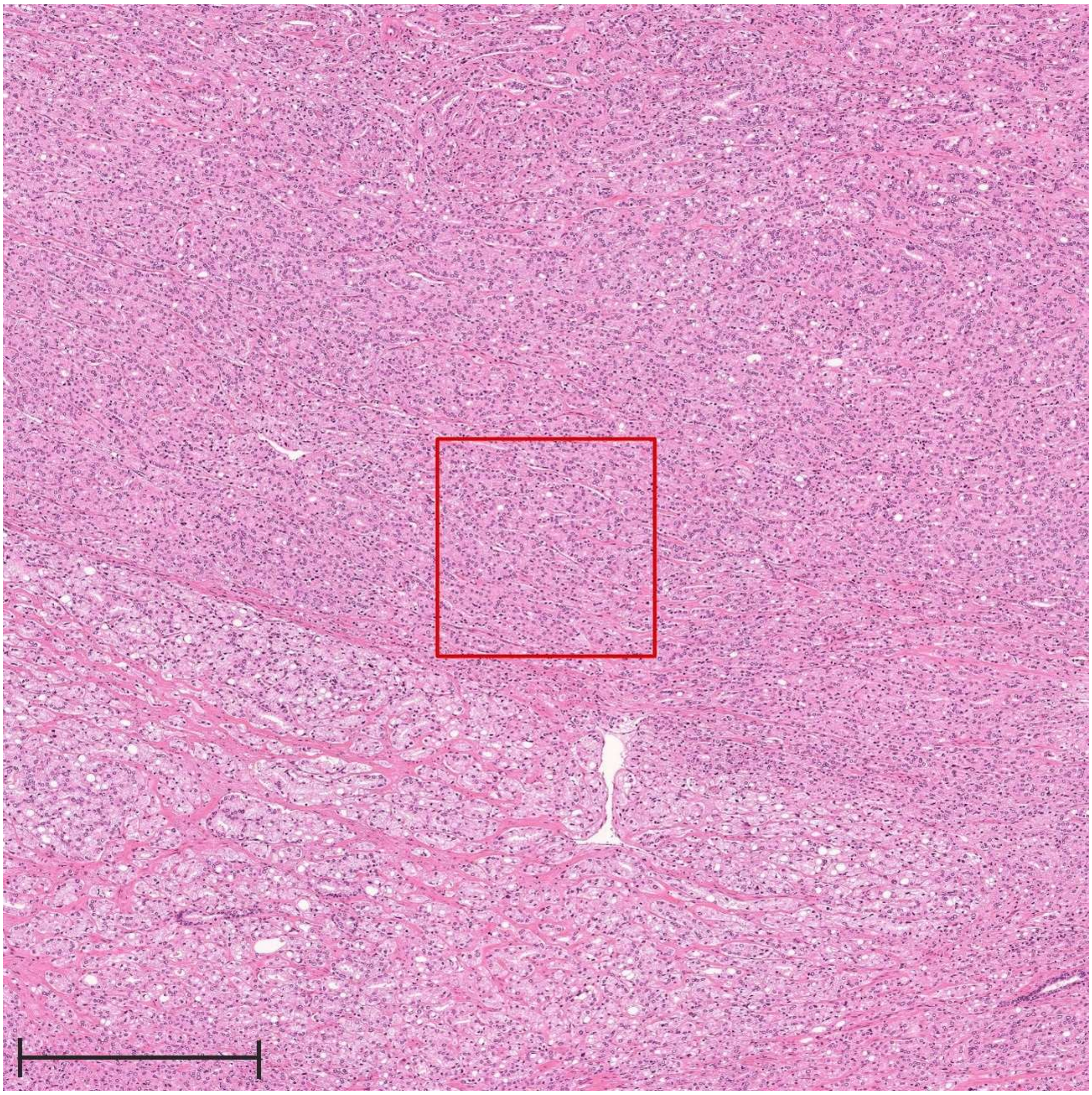

Supplementary Figure 32 | Representative image of a key feature (wide-range image).

Wide-range image contains a representative image of a weak predictor for biochemical recurrence. The representative image was detected by our algorithm and is indicated with a red square. The scale bar included in the image represents a length of 500 μm. Expert genitourinary pathologist's comments: Adenocarcinoma. The area is composed of adenocarcinoma cells, which is diagnosed as Gleason pattern 4.

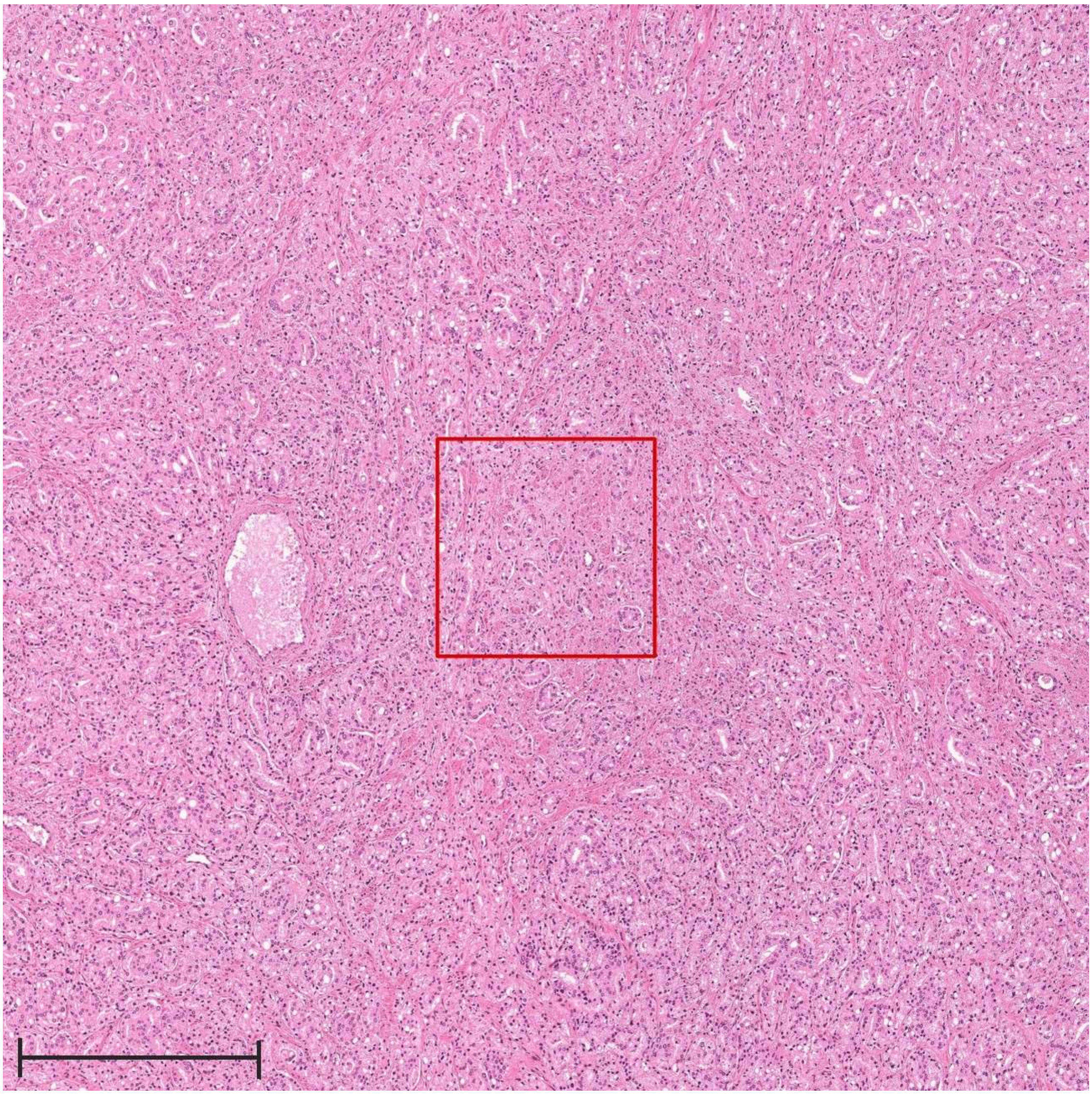

Supplementary Figure 33 | Representative image of a key feature (wide-range image).

Wide-range image contains a representative image of a weak predictor for biochemical recurrence. The representative image was detected by our algorithm and is indicated with a red square. The scale bar included in the image represents a length of 500 μm. Expert genitourinary pathologist's comments: Adenocarcinoma. The area is composed of adenocarcinoma cells, which is diagnosed as Gleason pattern 4.

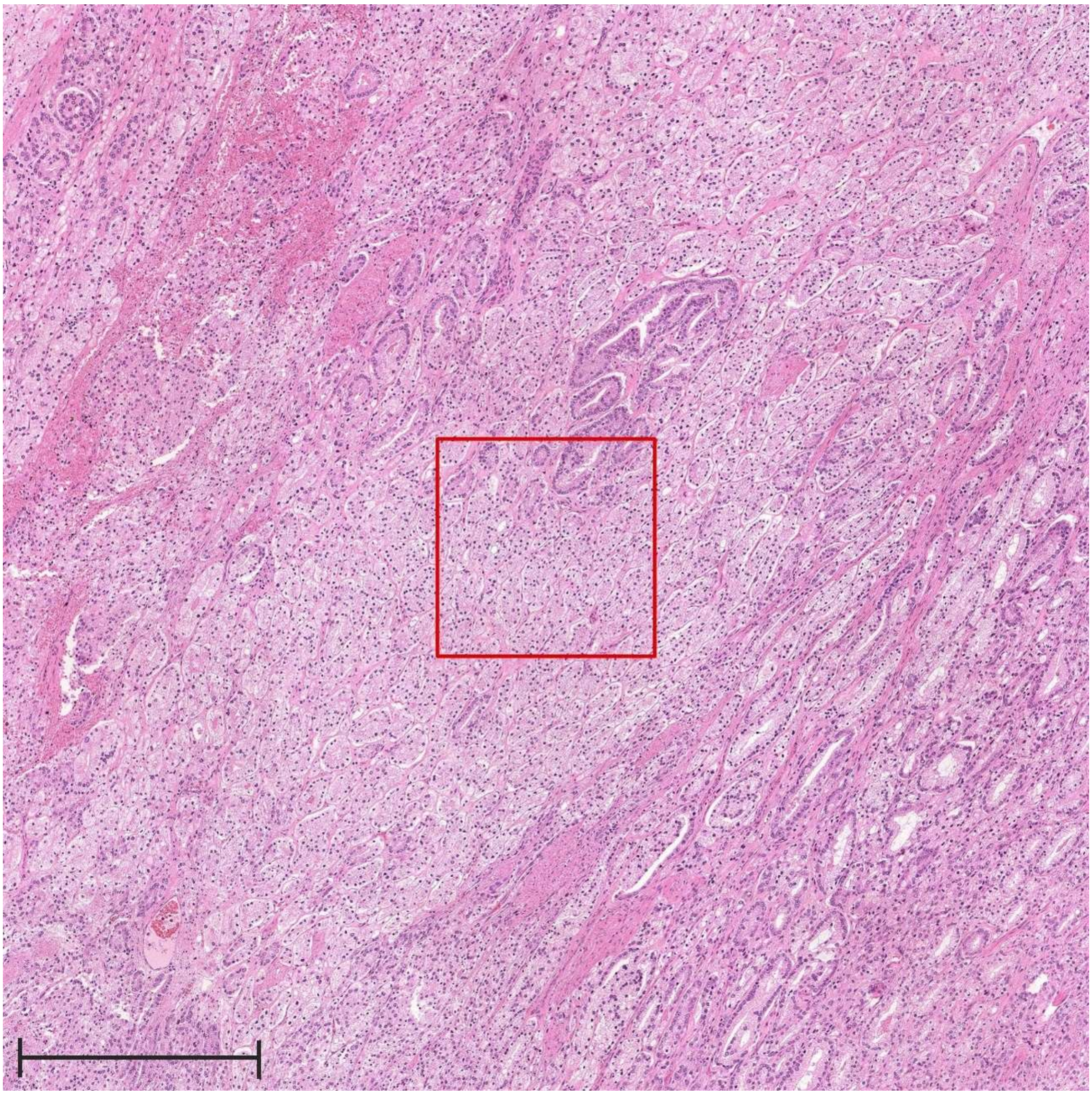

Supplementary Figure 34 | Representative image of a key feature (wide-range image).

Wide-range image contains a representative image of a weak predictor for biochemical recurrence. The representative image was detected by our algorithm and is indicated with a red square. The scale bar included in the image represents a length of 500 μm. Expert genitourinary pathologist's comments: Adenocarcinoma. The area shows adenocarcinoma cells, which is predominantly composed of Gleason pattern 4 (subsequently Gleason pattern 3).

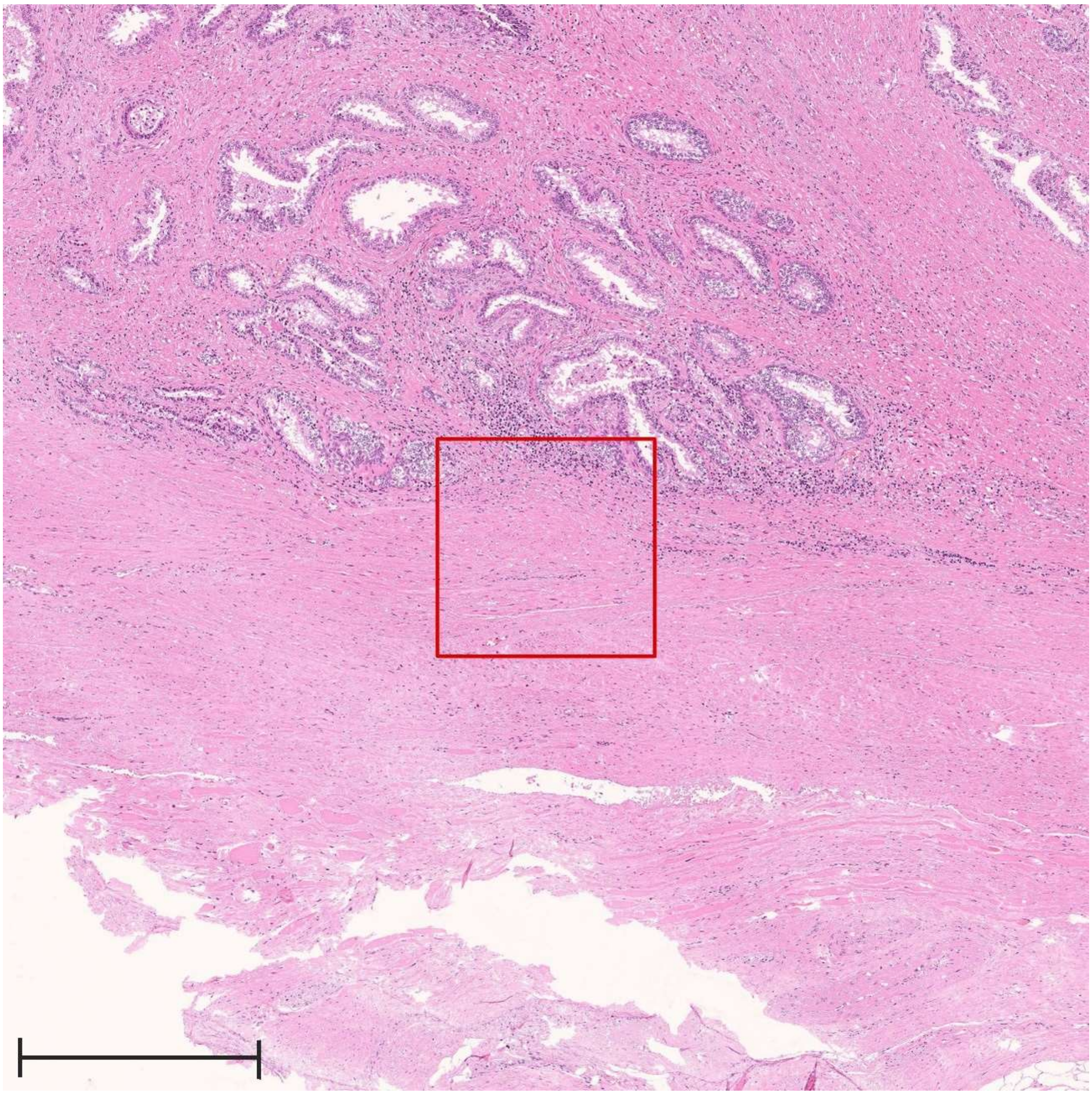

Supplementary Figure 35 | Representative image of a key feature (wide-range image).

Wide-range image contains a representative image of a weak predictor for biochemical recurrence. The representative image was detected by our algorithm and is indicated with a red square. The scale bar included in the image represents a length of 500 µm. Expert genitourinary pathologist's comments: Stromal component with small lymphocytes aggregation.

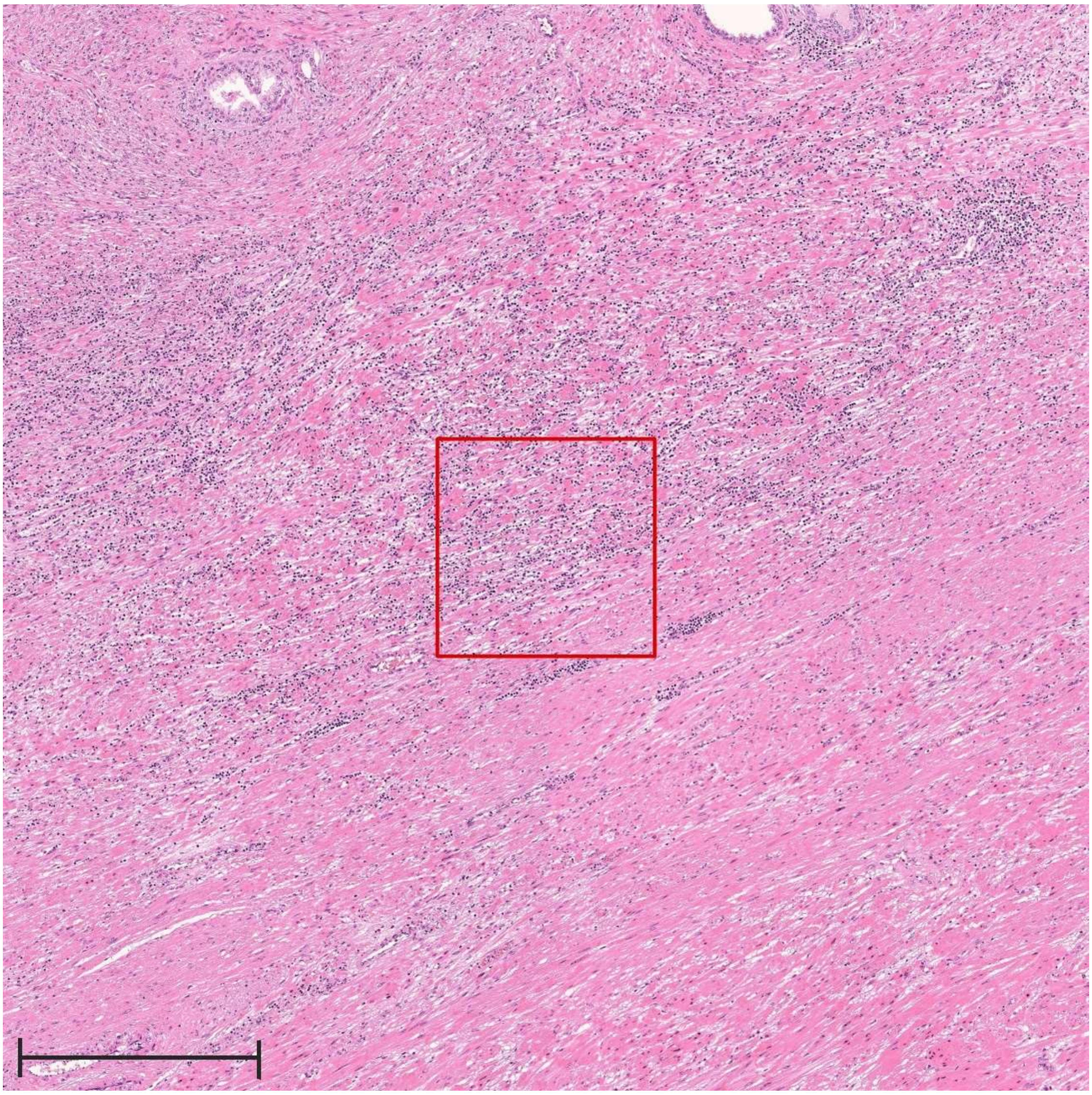

Supplementary Figure 36 | Representative image of a key feature (wide-range image).

Wide-range image contains a representative image of a weak predictor for biochemical recurrence. The representative image was detected by our algorithm and is indicated with a red square. The scale bar included in the image represents a length of 500 µm. Expert genitourinary pathologist's comments: Stromal component with small lymphocytes infiltration.

	BCR* (within 5 years) n=184	No BCR* (within 5 years) n=658	*P*-value
**Mean age, years (SD, range)**	66.3 (6.1, 49–81)	66.9 (5.9, 41–79)	0.25
**Mean height, cm (SD, range)**	166 (6.3, 147–185)	166 (5.7, 150–194)	0.61
**Mean weight, kg (SD, range)**	66.0 (9.8, 42–103)	64.8 (11, 40–193)	0.25
**Gleason score (≥ 8) / n (%)**	113/184 (61%)	175/658 (27%)	1.0×10^{-17}
**Mean PSA**, ng/mL (SD, range)**	22.8 (29, 3–218.9)	10.2 (9.9, 0.6–132.3)	2.1×10^{-22}
**Mean prostate weight, g (SD, range)**	46.3 (19, 11–142)	46.1 (17, 10–132)	0.95
**Clinical recurrence / n (%)**	22/184 (12%)	1/658 (0.2%)	2.0×10^{-14}

Supplementary Table 1. The clinical characteristics of Nippon Medical School Hospital dataset (NMSH) (5 years).

We compared the characteristics of patients whose cancer did or did not recur using the Fisher's exact test for categorical data and the Wilcoxon rank-sum test for continuous data.

* Biochemical recurrence (BCR).

** Prostate-specific antigen (PSA).

	AUC*	Pseudo R-squared
Gleason score (pathologist)	0.695 [95% CI 0.639–0.750]	0.137 [p-value 0.031]
Ridge (automated)	0.696 [95% CI 0.647–0.744]	0.133 [p-value 0.019]
Lasso (automated)	0.684 [95% CI 0.634–0.734]	0.122 [p-value 0.024]
SVM (automated)	**0.721 [95% CI 0.672–0.769]**	**0.141 [p-value 0.0099]**
Ridge + Gleason score	0.732 [95% CI 0.684–0.780]	0. 179 [p-value 0.0036]
Lasso + Gleason score	0.735 [95% CI 0.688–0.783]	0.180 [p-value 0.0067]
SVM + Gleason score	**0.758 [95% CI 0.710–0.806]**	**0.204 [p-value 0.0067]**

Supplementary Table 2. Comparison of biochemical recurrence (BCR) within 5 years in Nippon Medical School Hospital dataset (NMSH) (cross validation).

The reported values are averages with 95% confidence interval. The bold values indicate the highest accuracies for lasso, ridge and SVM.

* Area under the curve (AUC).

St. Marianna University Hospital (SMH)

	BCR* (within 1 year) n=5	No BCR* (within 1 year) n=47	*P*-value
**Mean age, years (SD, range)**	64.0 (3.8, 58–68)	67.7 (4.5, 57–75)	0.096
**Gleason score (≥ 8) / n (%)**	3/5 (60%)	16/47 (34%)	0.34
**Mean PSA**, ng/mL (SD, range)**	21.8 (8.2, 12–30.57)	10.4 (6.3, 4–29.4)	0.0043

Aichi Medical University Hospital (AMH)

	BCR* (within 1 year) n=3	No BCR* (within 1 year) n=40	*P*-value
**Mean age, years (SD, range)**	68.0 (5.6, 62–73)	69.5 (5.8, 51–80)	0.62
**Gleason score (≥ 8) / n (%)**	2/3 (67%)	14/40 (35%)	0.54
**Mean PSA**, ng/mL (SD, range)**	18.3 (8.5, 8.45–23.5)	9.90 (7.2, 4.2–36.9)	0.074

Supplementary Table 3. The clinical characteristics of external validation datasets.

We compared the characteristics of patients whose cancer did or did not recur using the Fisher's exact test for categorical data and the Wilcoxon rank-sum test for continuous data.

* Biochemical recurrence (BCR).

** Prostate-specific antigen (PSA).

	AUC*	Pseudo R-squared
Gleason score (pathologist)	0.712 [95% CI 0.492–0.931]	0.118 [p-value 0.072]
Ridge (automated)	0.837 [95% CI 0.729–0.945]	0.287 [p-value 0.0041]
Lasso (automated)	0.815 [95% CI 0.684–0.946]	0.252 [p-value 0.0074]
SVM (automated)	**0.841 [95% CI 0.732–0.949]**	**0.296 [p-value 0.0035]**
Ridge + Gleason score	0.859 [95% CI 0.740–0.977]	0.331 [p-value 0.0019]
Lasso + Gleason score	**0.891 [95% CI 0.770–1.000]**	0.317 [p-value 0.0024]
SVM + Gleason score	0.866 [95% CI 0.765–0.967]	**0.343 [p-value 0.0015]**

Supplementary Table 4. Comparison of biochemical recurrence (BCR) within 1 year at St. Marianna University Hospital (SMH) (external validation).

The reported values are averages with 95% confidence interval. The bold values indicate the highest accuracies for lasso, ridge and SVM.

* Area under the curve (AUC).

	AUC*	Pseudo R-squared
Gleason score (pathologist)	0.793 [95% CI 0.599–0.987]	0.168 [p-value 0.13]
Ridge (automated)	0.805 [95% CI 0.678–0.932]	0.178 [p-value 0.12]
Lasso (automated)	0.829 [95% CI 0.531–1.000]	0.163 [p-value 0.13]
SVM (automated)	**0.854 [95% CI 0.644–1.000]**	**0.202 [p-value 0.094]**
Ridge + Gleason score	0.915 [95% CI 0.828–1.000]	**0.321 [p-value 0.033]**
Lasso + Gleason score	0.902 [95% CI 0.805–1.000]	0.317 [p-value 0.034]
SVM + Gleason score	**0.927 [95% CI 0.839–1.000]**	0.297 [p-value 0.040]

Supplementary Table 5. Comparison of biochemical recurrence (BCR) within 1 year at Aichi Medical University Hospital (AMH) (external validation)

The reported values are averages with 95% confidence interval. The bold values indicate the highest accuracies for lasso, ridge and SVM.

* Area under the curve (AUC).

	BCR* (within 1 year)			
	**10 features [95% CI]**	**50 features [95% CI]**	**100 features [95% CI]**	**200 features [95% CI]**
Gleason score (pathologist)	0.743 [CI 0.665-0.821]	0.747 [CI 0.669-0.825]	0.744 [CI 0.672-0.816]	0.743 [CI 0.665-0.821]
Ridge (automated)	0.795 [CI 0.742–0.849]	0.801 [CI 0.744–0.858]	0.801 [CI 0.748–0.854]	0.789 [CI 0.726–0.853]
Lasso (automated)	0.791 [CI 0.734–0.849]	0.793 [CI 0.731–0.855]	0.804 [CI 0.749–0.860]	0.777 [CI 0.712–0.841]
SVM (automated)	0.798 [CI 0.742–0.854]	0.788 [CI 0.726–0.850]	0.820 [CI 0.766–0.873]	0.728 [CI 0.659–0.797]
Ridge + Gleason score	0.818 [CI 0.757–0.879]	0.821 [CI 0.763–0.880]	0.824 [CI 0.770–0.878]	0.797 [CI 0.730–0.865]
Lasso + Gleason score	0.812 [CI 0.745–0.878]	0.819 [CI 0.753–0.885]	0.830 [CI 0.772–0.888]	0.807 [CI 0.737–0.877]
SVM + Gleason score	0.816 [CI 0.752–0.880]	0.794 [CI 0.727–0.860]	0.842 [CI 0.788–0.896]	0.748 [CI 0.679–0.817]

Supplementary Table 6. AUC comparison amoung different feature sets in NMSH dataset (cross-validation).

The reported values are averages with 95% confidence interval.

*Biochemical recurrence (BCR).
