# Supplementary material for: Automated acquisition of explainable knowledge from unannotated histopathology images
Source: Nat Commun. 2019 Dec 18;10:5642. doi: 10.1038/s41467-019-13647-8 (PMC6920352; doi:10.1038/s41467-019-13647-8)
Supplement: Supplementary file 2 — Description of Additional Supplementary Files [file 41467_2019_13647_MOESM2_ESM.docx]

**Description of Additional Supplementary Files**

**File Name: Supplementary Movie 1**

**Description:** The movie shows an automatically annotated wholemount pathology image (left), as well as a low-magnification image of the yellow region (upper right) and the associated high-magnification images with number of Step 2 feature (lower right). The regions with impact scores above and below 0.5 in Step 1 are shaded in red and blue, respectively. The indicated yellow cell shows [number of Step 1 feature (100 total features)] [impact score, Step 1] [impact score, Step 2] (see Key feature generation method in the methods section). Cells that belong to the same feature as the yellow cell are simultaneously shaded. In this movie, cancer occupies the transitional zone of prostate. The outer region and the inner region of cancer are classified into distinct features.

**File Name: Supplementary Movie 2**

**Description:** The movie shows an automatically annotated wholemount pathology image (left), as well as a low-magnification image of the yellow region (upper right) and the associated high-magnification images with number of Step 2 feature (lower right). The regions with impact scores above and below 0.5 in Step 1 are shaded in red and blue, respectively. The indicated yellow cell shows [number of Step 1 feature (100 total features)] [impact score, Step 1] [impact score, Step 2] (see Key feature generation method in the methods section). Cells that belong to the same feature as the yellow cell are simultaneously shaded. In this movie, cancer broadly occupies the transitional zone to the peripheral zone of prostate. Different types of cancer exist heterogeneously.
