# Supplementary material for: Automated acquisition of explainable knowledge from unannotated histopathology images
Source: Nat Commun. 2019 Dec 18;10:5642. doi: 10.1038/s41467-019-13647-8 (PMC6920352; doi:10.1038/s41467-019-13647-8)
Supplement: Supplementary file 5 — Reporting Summary [file 41467_2019_13647_MOESM5_ESM.pdf]

nature research

Corresponding author(s): Yoichiro Yamamoto, Go kimura

Last updated by author(s): Nov 18, 2019

Reporting Summary

Nature Research wishes to improve the reproducibility of the work that we publish. This form provides structure for consistency and transparency in reporting. For further information on Nature Research policies, see Authors & Referees and the Editorial Policy Checklist .

Statistics

For all statistical analyses, confirm that the following items are present in the figure legend, table legend, main text, or Methods section.

n/a	Confirmed	
☐	☒	The exact sample size (*n*) for each experimental group/condition, given as a discrete number and unit of measurement
☐	☒	A statement on whether measurements were taken from distinct samples or whether the same sample was measured repeatedly
☐	☒	The statistical test(s) used AND whether they are one- or two-sided<br>*Only common tests should be described solely by name; describe more complex techniques in the Methods section.*
☐	☒	A description of all covariates tested
☐	☒	A description of any assumptions or corrections, such as tests of normality and adjustment for multiple comparisons
☐	☒	A full description of the statistical parameters including central tendency (e.g. means) or other basic estimates (e.g. regression coefficient) AND variation (e.g. standard deviation) or associated estimates of uncertainty (e.g. confidence intervals)
☐	☒	For null hypothesis testing, the test statistic (e.g. *F*, *t*, *r*) with confidence intervals, effect sizes, degrees of freedom and *P* value noted<br>*Give P values as exact values whenever suitable.*
☒	☐	For Bayesian analysis, information on the choice of priors and Markov chain Monte Carlo settings
☒	☐	For hierarchical and complex designs, identification of the appropriate level for tests and full reporting of outcomes
☐	☒	Estimates of effect sizes (e.g. Cohen's *d*, Pearson's *r*), indicating how they were calculated

Our web collection on statistics for biologists contains articles on many of the points above.

Software and code

Policy information about availability of computer code

Data collection	We analyzed all patients (1,007) who received radical prostatectomy between April 2000 and December 2016 at the Nippon Medical School Hospital (NMSH). For external validation, we analyzed all patients who received radical prostatectomy from August 2013 to August 2017 at the St. Marianna University Hospital (SMH) (N = 55) and from January 2016 to June 2016 at the Aichi Medical University Hospital (AMH) (N = 47). This research has been approved by each Institutional Review Board (IRB): NMSH (reference 28-11-663), SMH (reference 3887), AMH (reference 2019-H045) and RIKEN (reference Wako3 29-14).
Data analysis	We developed a new method of generating key features from images that employs two different unsupervised deep neural networks (deep autoencoders) at different magnifications and weighted non-hierarchical clustering (see Methods section and Supplementary Figures). Analysis was performed with custom code written in Python. In Key feature generation, we presented our detailed algorithm flowchart, networks of deep autoencoders and the methods in this paper instead of full source code. All processes are described in enough detail to enable independent replication and the full source code were submitted for review process. All software (packages for R) in BCR predictions for evaluation of generated features are publicly available: the glmnet package (version 2.0.16), the e1071 package (version 1.7.0), cvAUC package (version 1.1.0), pROC package (version 1.13.0) and rcompanion package (version 2.2.1).

For manuscripts utilizing custom algorithms or software that are central to the research but not yet described in published literature, software must be made available to editors/reviewers. We strongly encourage code deposition in a community repository (e.g. GitHub). See the Nature Research guidelines for submitting code & software for further information.

Data

Policy information about availability of data

All manuscripts must include a data availability statement. This statement should provide the following information, where applicable:

- Accession codes, unique identifiers, or web links for publicly available datasets
- A list of figures that have associated raw data
- A description of any restrictions on data availability

The clinical datasets used were collected at the NMSH, SMH and AMH. This work and the collection of data was approved by the Institutional Review Board of each

hospital. They are not publicly available, and restrictions apply to their use.

Field-specific reporting

Please select the one below that is the best fit for your research. If you are not sure, read the appropriate sections before making your selection.

- [x] Life sciences
- [ ] Behavioural & social sciences
- [ ] Ecological, evolutionary & environmental sciences

For a reference copy of the document with all sections, see nature.com/documents/nr-reporting-summary-flat.pdf

Life sciences study design

All studies must disclose on these points even when the disclosure is negative.

Sample size	NMSH dataset: we analyzed all patients with prostate cancers (1,007) who received radical prostatectomy between April 2000 and December 2016 at the Nippon Medical School Hospital.<br>External validation datasets: we analyzed all patients who received radical prostatectomy from August 2013 to August 2017 at the St. Marianna University Hospital (SMH) (N = 55) and from January 2016 to June 2016 at the Aichi Medical University Hospital (AMH) (N = 47).
Data exclusions	NMSH dataset: we excluded 115 cases involving neoadjuvant therapy and 7 cases involving adjuvant therapy as well as 43 cases who could not be followed up within 1 year because of hospital transfer or death due to other causes, thus leaving 842 cases for analysis.<br>External validation datasets: we excluded 1 case involving neoadjuvant therapy and 1 case because of missing slides as well as 5 cases who could not be followed up within 1 year because of hospital transfer, thus leaving 95 cases for analysis.
Replication	We conducted external validation of cancer recurrence predictions based on datasets from SMH and AMH.
Randomization	We used all patient data without bias.
Blinding	Investigators of the evaluation of prediction models were blinded to diagnostic result and were not involved in dataset collection.

Reporting for specific materials, systems and methods

We require information from authors about some types of materials, experimental systems and methods used in many studies. Here, indicate whether each material, system or method listed is relevant to your study. If you are not sure if a list item applies to your research, read the appropriate section before selecting a response.

Materials & experimental systems

n/a	Involved in the study	
x		Antibodies
x		Eukaryotic cell lines
x		Palaeontology
x		Animals and other organisms
x		Human research participants
	x	Clinical data

Methods

n/a	Involved in the study	
x		ChIP-seq
x		Flow cytometry
x		MRI-based neuroimaging

Clinical data

Policy information about clinical studies

All manuscripts should comply with the ICMJE guidelines for publication of clinical research and a completed CONSORT checklist must be included with all submissions.

Clinical trial registration	This study is not a clinical trial.
Study protocol	We predicted cancer recurrence using deep learning-generated features. We used AUC and pseudo R-squared for comparison of deep learning-generated features and Gleason score. Please see "Method" in our manuscript.
Data collection	NSMH dataset: We used all patients with prostate cancers (1,007) who received radical prostatectomy between April 2000 and December 2016 at the Nippon Medical School Hospital. We excluded 115 cases involving neoadjuvant therapy and 7 cases involving adjuvant therapy as well as 43 cases who could not be followed up within 1 year because of hospital transfer or death due to other causes, thus leaving 842 cases for analysis. We found that cancer was more likely to recur in patients with higher prostate-specific antigen (PSA) levels (P-value < 0.001). It was more likely to recur in patients with higher Gleason scores (≥ 8) than in patients with lower Gleason scores (< 8). Similar patterns were observed for 1-year and 5-year recurrence rates. No significant differences existed in the average age, height, weight, or prostate weight between patients in whom cancer recurred and those in whom it did not.<br>External validation datasets: we analyzed all patients who received radical prostatectomy from August 2013 to August 2017 at SMH (N = 55) and from January 2016 to June 2016 at the AMH (N = 47). We excluded 1 case involving neoadjuvant therapy and 1 case because of missing slides as well as 5 cases who could not be followed up within 1 year because of hospital transfer, thus leaving 95 cases for analysis. Only PSA level of patients with recurrent cancer at SMH was significantly higher than that of non-recurrent patients (P-value = 0.0043).
Outcomes	Biochemical recurrence
